# ACE mRNA (Additional Chimeric Element incorporated IVT mRNA) for Enhancing Protein Expression by Modulating Immunogenicity

**DOI:** 10.1002/advs.202307541

**Published:** 2024-03-06

**Authors:** Sora Son, Minsa Park, Jin Kim, Kyuri Lee

**Affiliations:** ^1^ College of Pharmacy and Research Institute of Pharmaceutical Sciences Gyeongsang National University Jinju Gyeongsangnam‐do 52828 Republic of Korea

**Keywords:** immunogenicity, mRNA therapeutics, protein expression, RNA structure

## Abstract

The development of in vitro transcribed mRNA (IVT mRNA)‐based therapeutics/vaccines depends on the management of IVT mRNA immunogenicity. IVT mRNA, which is used for intracellular protein translation, often triggers unwanted immune responses, interfering with protein expression and leading to reduced therapeutic efficacy. Currently, the predominant approach for mitigating immune responses involves the incorporation of costly chemically modified nucleotides like pseudouridine (Ψ) or N1‐methylpseudouridine (m1Ψ) into IVT mRNA, raising concerns about expense and the potential misincorporation of amino acids into chemically modified codon sequences. Here, an Additional Chimeric Element incorporated mRNA (ACE mRNA), a novel approach incorporating two segments within a single IVT mRNA structure, is introduced. The first segment retains conventional IVT mRNA components prepared with unmodified nucleotides, while the second, comprised of RNA/DNA chimeric elements, aims to modulate immunogenicity. Notably, ACE mRNA demonstrates a noteworthy reduction in immunogenicity of unmodified IVT mRNA, concurrently demonstrating enhanced protein expression efficiency. The reduced immune responses are based on the ability of RNA/DNA chimeric elements to restrict retinoic acid‐inducible gene I (RIG‐I) and stimulator of interferon genes (STING)‐mediated immune activation. The developed ACE mRNA shows great potential in modulating the immunogenicity of IVT mRNA without the need for chemically modified nucleotides, thereby advancing the safety and efficacy of mRNA‐based therapeutics/vaccines.

## Graphical abstract

The Additional Chimeric Element incorporated mRNA (ACE mRNA) represents a breakthrough in reducing immunogenicity of in vitro transcribed mRNA (IVT mRNA). ACE mRNA incorporates two segments within a single structure, the first retaining conventional components and the second consisting of RNA/DNA chimeric elements. This innovative structure demonstrates reduced immunogenicity, thereby enhancing protein expression without the need for chemically modified nucleotides.

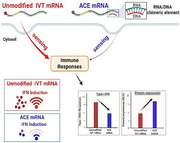

## 1 Introduction

Discovering approaches to modulate the immunostimulatory effects of in vitro transcribed mRNA (IVT mRNA) is crucial for the successful development of mRNA‐based therapeutics/vaccines.^[^ ^1^, ^2^ ^]^ Within clinical contexts, IVT mRNA is synthesized to mediate translation processes, leading to the production of target proteins within cells. Upon intracellular delivery, IVT mRNA generates innate immune responses by being recognized by pattern recognition receptors (PRRs) as non‐self RNAs.^[^ ^3^, ^4^ ^]^ These immune responses act as an inhibitory signal on the cellular translation machinery, consequently reducing IVT mRNA‐mediated protein expression efficiency. Like this, there is an inverse relationship between immune responses and translation.^[^ ^5^ ^]^ The required balance between immune responses and translation may differ depending on the specific aims of clinical applications. In vaccine development, it is not optimal to completely eliminate the immunogenicity of IVT mRNA. Instead, the focus should be on a partial reduction to a level that guarantees translation‐dependent antigen presentation. This strategic approach is guided by the importance of maintaining appropriate levels of both translation and immune responses for vaccine efficacy.^[^ ^6^ ^]^ On the other hand, mRNA‐based therapeutics such as protein replacement therapy primarily necessitate translation for sufficient protein expression without triggering any immune responses.^[^ ^7^ ^]^ In this regard, the primary goal of mRNA‐based therapeutics/vaccines is to develop a range of strategies aimed at reducing the excessive immunostimulatory effects of IVT mRNA to varying degrees for different clinical applications.^[^ ^8^, ^9^ ^]^

Cells possess various types of PRRs that have the potential to be activated by IVT mRNA.^[^ ^10^, ^11^ ^]^ These include Toll‐like receptors (TLRs) such as TLR3,^[^ ^12^ ^]^ TLR7,^[^ ^13^, ^14^ ^]^ and TLR8,^[^ ^15^, ^16^ ^]^ as well as the RIG‐I‐like receptor family, which consists of retinoic acid‐inducible gene I (RIG‐I)^[^ ^17^, ^18^ ^]^ and melanoma differentiation‐associated gene 5 (MDA5).^[^ ^19^, ^20^ ^]^ Each PRR has its own ability to discriminate between specific types of nucleic acid structures, relying on its distinct pathogen‐associated molecular patterns (PAMPs) and chemical modification. Upon sensitization, these PRRs initiate immune responses by inducing transcription factors such as nuclear factor‐κB (NF‐κB) and interferon (IFN)‐regulatory factor 3 (IRF3).^[^ ^21^, ^22^ ^]^ To reduce the immunostimulatory effect of IVT mRNA, it is necessary to bypass the recognition and activation pathway mediated by PRRs. By altering the PAMPs or chemical modification patterns of IVT mRNA, the PRRs‐mediated recognition of non‐self RNAs and subsequent activation processes of immune responses can be modulated, resulting in different levels of immune stimulation that affect the protein expression efficiency.^[^ ^23^ ^]^

The current primary approach for modulating the immunostimulatory effect of IVT mRNA involves incorporating chemically modified nucleotides, specifically pseudouridine (Ψ) or N1‐methylpseudouridine (m1Ψ), into the IVT mRNA structure.^[^ ^8^, ^24^ ^]^ The incorporation of Ψ or m1Ψ into IVT mRNA has demonstrated reduced immunostimulatory effects as they bypass TLR7‐mediated recognition of single‐stranded RNA (ssRNA) containing unmodified uridine.^[^ ^25^ ^]^ This leads to a significant improvement in target protein production by almost completely eliminating immune responses and serves as a key technology in the clinical application of IVT mRNA. Nevertheless, despite these advancements, chemically modified IVT mRNA encounters a number of unresolved challenges: 1) limited technical options to modulate the immunogenic properties of IVT mRNAs to varying degrees for a variety of clinical applications,^[^ ^26^, ^27^ ^]^ 2) the high cost of manufacturing, which hampers the broad accessibility of mRNA therapeutics/vaccines, particularly in economically disadvantaged countries,^[^ ^28^ ^]^ and 3) the potential risk of misreading of codons in the chemically modified IVT mRNA during the translation process, resulting in the generation of misfolded proteins with reduced or even no therapeutic efficacy.^[^ ^29^, ^30^ ^]^ To overcome these limitations, there is a strong need to develop a strategy to modulate the immunogenicity of unmodified IVT mRNA without the need for a chemically modified nucleotide.

Here, we developed a novel type of IVT mRNA structure incorporating two distinct segments within a single IVT mRNA structure. The first segment remains consistent with the conventional form of unmodified IVT mRNA including 5′ cap, 5′ untranslated region (UTR), open reading frame (ORF), 3′ UTR, and poly(A) tail, and the second segment is an additional compartment designed to have the potential to modulate immunogenicity of the IVT mRNA. There have been several reports showing that some types of non‐canonical nucleic acid structures can reduce immune responses by bypassing the recognition and activation of PRRs.^[^ ^25^, ^31^, ^32^ ^]^ Based on this knowledge, it was hypothesized that by incorporating such non‐canonical nucleic acid structure into the IVT mRNA as the second segment, the immunogenicity of unmodified IVT mRNA could be controlled. Despite the potential, there have been limited attempts to incorporate such nucleic acid structures into IVT mRNA due to the difficulty of incorporating them while preserving the translational capacity of the IVT mRNA. To address this issue, we have engineered IVT mRNA structures to enable the introduction of the non‐canonical nucleic acid structure into IVT mRNA without affecting its essential role in translation. Specifically, RNA/DNA chimeric element was introduced at the 3′ ends of the unmodified IVT mRNA, downstream the poly(A) tail, resulting in the formation of an Additional Chimeric Element incorporated mRNA (ACE mRNA). Several reports have shown that the RNA/DNA chimeric element can potentially restrict RNA recognition and subsequent activation of immune responses.^[^ ^33^, ^34^ ^]^ In this regard, it was anticipated that the introduced RNA/DNA chimeric structure would function as an additional compartment to mitigate the immune responses of IVT mRNA. In addition, with regard to translational capacity, it was hypothesized that the chimeric element would not interfere with mRNA translation, as intracellular RNase H possesses the capability to remove RNA/DNA chimeric elements from IVT mRNAs. As expected, it was successfully verified that the developed ACE mRNA exhibits reduced immunogenicity and increased protein expression efficiency without requiring chemically modified nucleotides, circumventing the limitations of chemical modification techniques. Given that ACE mRNA is basically based on the unmodified IVT mRNA, the aforementioned limitation of the high cost of modified nucleotides and concerns about codon misinterpretation can be effectively addressed. Moreover, ACE mRNA holds promise as a versatile strategy for regulating the immunogenicity of IVT mRNAs, as the first segment can be customized to suit specific clinical objectives, with only the additional incorporation of the second segment influencing immunogenicity. Looking ahead, ACE mRNA offers a valuable strategy for modulating the immunogenicity of IVT mRNA, thereby circumventing the limitations associated with the current primary strategy of Ψ or m1Ψ chemical modification.

## 2 Results and Discussion

### 2.1 dsRNA By‐Product Purification Is Not Sufficient to Completely Reduce IVT mRNA‐Induced Type I IFN Responses

During the process of synthesizing IVT mRNA using in vitro transcription (IVT), the production of unintended by‐products in the form of double‐stranded RNA (dsRNA) is unavoidable. These dsRNA by‐products can activate innate immune responses, resulting in the production of type I IFN, which subsequently reduces the efficiency of protein expression. To eliminate these dsRNA by‐products, cellulose purification methods can be employed, which take advantage of the specific binding of dsRNA to cellulose under conditions involving an ethanol‐containing buffer. In more detail, the IVT mRNA is exposed to cellulose within spin columns under the 16% (v/v) ethanol‐containing buffered conditions.^[^ ^35^ ^]^ During this incubation, the dsRNA by‐products bind to the cellulose, while the ssRNA form is released as an unbound fraction. Therefore, the cellulose purification steps are highly required to manage the excessive immune stimulation induced by dsRNA by‐products of IVT mRNA.

**Figure 1:**
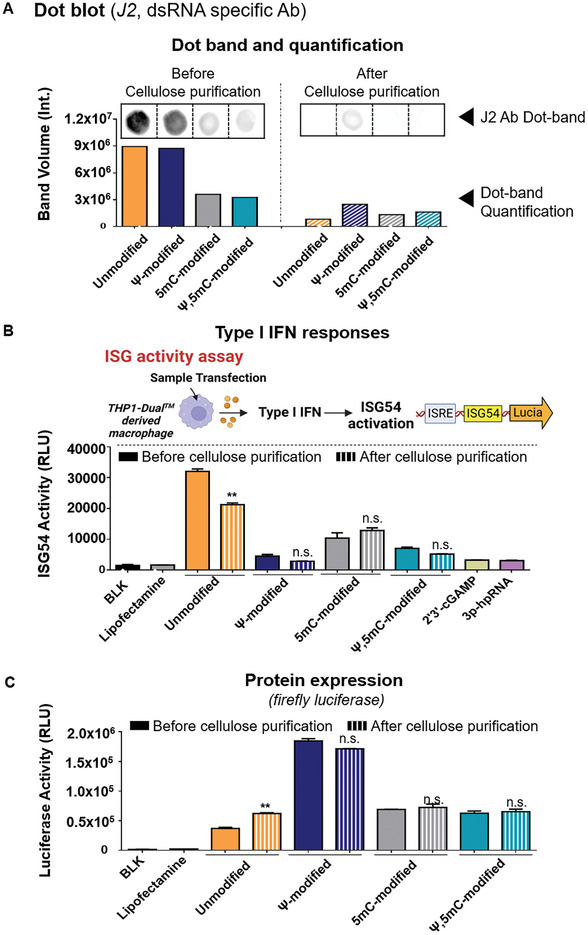
Detection and quantification of dsRNA by‐products in IVT mRNAs with various chemical modifications (unmodified, Ψ‐, 5mC‐, or Ψ, 5mC‐modified) before and after cellulose purification, as assessed using the J2 antibody. A) The presence of dsRNA by‐products in unmodified and chemically modified (Ψ‐, 5mC‐, or a Ψ, 5mC‐) IVT mRNA was detected using J2 antibodies before and after cellulose purification. Dot bands in the figure represent the detected dsRNA by‐products, and the bar graph illustrates the quantification of these dsRNA dot bands, presented as adjusted band volumes. B) Type I IFN responses in THP1‐Dual cells were assessed before and after cellulose purification of unmodified and chemically modified IVT mRNA (Ψ‐, 5mC‐, or a Ψ, 5mC‐) by measuring Lucia luciferase expression, indicating ISG54 activity. C) Protein expression efficiency before and after cellulose purification of unmodified or chemically modified IVT mRNA (Ψ‐, 5mC‐, or a Ψ, 5mC‐), verified by measuring firefly luciferase levels 24 h post‐transfection in HEK 293T cell line. Statistical significance was determined using a Student's *t*‐test. (ns: not significant and **p < 0.01).

Various IVT mRNAs encoding firefly luciferase were prepared using different combinations of chemically modified nucleotides (unmodified, Ψ‐modified, 5mC (5‐methylcytidine)‐modified or Ψ, 5mC‐modified) and the amount of dsRNA by‐products was assessed by dot blot analysis using the J2 antibody, which specifically detects dsRNA. In addition, ImageLab software was utilized to calculate the band volume intensities observed in the dot blot analysis (**Figure** **1**A). The IVT mRNA prepared with different combinations of chemically modified nucleotides showed varying degrees of band volume intensities. IVT mRNA synthesized with unmodified nucleotides (unmodified) and IVT mRNA in which unmodified uridine was replaced with Ψ (Ψ ‐modified) exhibited elevated levels of dsRNA by‐products. In contrast, IVT mRNA containing chemically modified nucleotides of 5mC (5mC‐modified) or a double modification of Ψ and 5mC (Ψ, 5mC‐modified) showed reduced levels of dsRNA. Upon comparing the quantity of dsRNA before and after cellulose purification, we observed a significant reduction in the band volume intensities in the dot blot analysis. This indicates that the cellulose purification method was effective in removing the dsRNA by‐products, regardless of the incorporation of chemically modified nucleotides.

To assess the impact of cellulose purification on the immune stimulation of IVT mRNA in vitro, THP1‐Dual cells were utilized. These cells are derived from the human THP‐1 monocyte cell line and have been engineered to allow for the simple analysis of the IRF pathway by measuring Lucia luciferase expression. The measured Lucia luciferase levels are indicative of ISG54 and IRF pathway activation, as the Lucia luciferase gene is under the control of the ISG54 minimal promoter. The IVT mRNA prepared with different chemical modifications and followed by with or without cellulose purification were treated to the THP1‐Dual cells using lipofectamine 2000. Subsequently, the levels of ISG54 activities were evaluated by measuring Lucia luciferase expression (Figure 1B). The results showed that IVT mRNA prepared with unmodified nucleotides induced a high level of ISG54 activities, indicating an elevated activation of the IRF pathway and production of type I interferon. On the other hand, the incorporation of chemically modified nucleotides (Ψ‐, 5mC‐, or a Ψ, 5mC‐modified) into IVT mRNA reduced the ISG54 activities, indicating less induction of type I interferon. In addition, the cellulose purification process played a role in diminishing the ISG54 activities of unmodified IVT mRNA, likely achieved through the removal of dsRNA by‐products. However, the cellulose purification exhibited a minimal effect on the reduction of ISG54 activities in IVT mRNAs prepared using chemically modified nucleotides (Ψ‐, 5mC‐, or a Ψ, 5mC‐modified IVT mRNA). Subsequently, the cellulose purification process significantly increased the protein expression efficiency of IVT mRNA prepared with unmodified nucleotides, while having a negligible effect on chemically modified IVT mRNA (Figure 1C). These results indicate that the cellulose purification process reduced the excessive immunostimulatory effect of unmodified IVT mRNAs by removing the dsRNA by‐products, resulting in enhanced protein expression efficiency. However, this effect was observed only in the case of unmodified IVT mRNAs, and there was minimal impact on IVT mRNAs with chemical modifications (Ψ‐, 5mC‐, or a Ψ, 5mC‐modified IVT mRNA). In addition, despite the beneficial impact of cellulose purification in improving protein expression with unmodified IVT mRNAs, the enhanced efficiency of protein expression was still insufficient compared to that achieved with Ψ‐modified IVT mRNAs.

### 2.2 Design and Preparation of Additional Chimeric Element Incorporated IVT mRNA (ACE mRNA)

Beyond chemical modification, IVT mRNAs can generate varying degrees of immune responses, primarily influenced by their distinct molecular patterns, which can be altered by modifying the sequences of IVT mRNAs. Nonetheless, modifying the sequence of IVT mRNAs presents challenges due to limited available options for introducing sequence variations. The sequence of the ORF region in IVT mRNAs is constrained to the amino acid sequences of the target protein they aim to produce. Additionally, the 5′ and 3′ UTR regions are also restricted to optimized sequences to achieve high efficiency in protein expression. As a hypothesis, it was suggested that incorporating chimeric elements into IVT mRNA could potentially influence the immune responses triggered by IVT mRNA. Nevertheless, the translation capabilities of IVT mRNA can be significantly reduced due to the presence of introduced chimeric elements. Here, we propose a novel approach called ACE mRNA, which involves additional RNA/DNA chimeric elements downstream of the poly(A) tail at the 3′ ends of IVT mRNA. This strategy aims to introduce chimeric elements that have the potential to modulate the immune responses of the IVT mRNA while maintaining or even enhancing their protein expression efficiency. It was anticipated that ACE mRNA would demonstrate the potential to provide an appropriate balance between immune responses and translation mediated by IVT mRNAs according to their clinical applications, without the chemical modification techniques.

In general, the DNA template used for IVT mRNA preparation includes a T7 promoter for T7 polymerase‐mediated in vitro transcription (IVT) reaction, as well as the 5′ UTR, ORF, 3′ UTR, and can include poly(dA:dT) for poly(A) tailing. To introduce RNA/DNA chimeric elements at the 3′ ends of IVT mRNA, additional sequences (sequence B or C) were introduced downstream of the poly(dA:dT) within the DNA template through a polymerase chain reaction (PCR) process (**Figure** **2**A). In the PCR procedure, reverser primers containing poly(dT) and additional sequences (sequence B or C) were utilized to introduce poly(dA:dT), and additional sequences (sequence B or C) into PCR products. This resulted in the preparation of PCR products including T7 promoter, 5′ UTR, ORF, 3′ UTR, poly(dA:dT), and additional sequences (sequence B or C). The purpose of including poly(dA:dT) and the additional sequences in the PCR products was to introduce the poly(A) tail and RNA/DNA chimeric elements into IVT mRNAs. The additional sequences (sequence B or C) were specifically designed for the annealing process with DNA oligos, which facilitated the incorporation of the RNA/DNA chimeric elements. Sequence B contains additional sequences that are shorter in length (21 base pairs (bp)), while sequence C has a longer sequence length (51 bp). The prepared PCR products were used as a DNA template for IVT reaction to synthesize IVT mRNAs. The synthesized IVT mRNA, which now included the additional sequences B or C downstream of the poly(A) tail, was purified using a cellulose purification process to eliminate dsRNA by‐products. Subsequently, DNA oligos with complementary sequences to the introduced additional sequences in the IVT mRNA were annealed into the IVT mRNA by a slow annealing process. This resulted in the incorporation of RNA/DNA chimeric elements at the 3′ ends of the IVT mRNA, with these elements being specifically designed to have a 2 nt RNA overhang. The annealed DNA oligos also include additional dT sequences (6 dT for sequence B and 11 dT for sequence C) that can be annealed to a portion of poly(A) tail in addition to the additional sequences (sequence B or C) introduced in the prepared IVT mRNAs. Throughout this process, two variations of ACE mRNA were prepared: mRNA/DNA25B and mRNA/DNA60C, containing 25 and 60 bp of RNA/DNA chimeric elements with a 2 nt RNA overhang, respectively, located at the 3′ ends of IVT mRNA.

Figure 2Additional Chimeric Element incorporated IVT mRNA (ACE mRNA). A) Schematic illustration of design and preparation of Additional Chimeric Element incorporated mRNA (ACE mRNA), incorporating RNA/DNA chimeric elements downstream of the poly(A) tail at the 3′ ends of IVT mRNA. PCR products containing the T7 promoter, 5′ UTR, ORF, 3′ UTR, poly(dA:dT), and additional sequences (sequence B or C) served as the DNA template for IVT mRNA synthesis. DNA oligos with sequences complementary to the introduced additional sequences (sequence B or C) in IVT mRNA were slowly annealed into the IVT mRNA, resulting in the creation of RNA/DNA chimeric element incorporated mRNA (ACE mRNA). B) Confirmation of the successful introduction of RNA/DNA chimeric elements into ACE mRNA. In the polyacrylamide gel electrophoresis assay, the bands corresponding to DNA oligos (DNA25: complementary to sequence B + 6 dT, DNA60: complementary to sequence C + 11 dT) disappeared after the slow annealing process with IVT mRNA containing sequence B or C. The mRNA integrity of ACE mRNA was further verified by 1% agarose electrophoresis assay. C) Verification of successful introduction of RNA/DNA chimeric elements into ACE mRNA. The presence of RNA/DNA chimeric elements within the IVT mRNA was detected using the S9.6 antibody, which can differentiate RNA/DNA structures. D) Evaluation of type I IFN responses of ACE mRNA compared to various mRNA constructs, assessed in THP1‐Dual cells. The levels of type I IFN induction by two ACE mRNA variants (mRNA/DNA25B, mRNA/DNA60C) were compared with Ψ‐modified IVT mRNA, unmodified IVT mRNA, short RNA/DNA with the same sequence and length as the RNA/DNA chimeric elements introduced into either mRNA/DNA25B or mRNA/DNA60C (RNA/DNA25B, RNA/DNA60C), and a simple mixture of unmodified IVT mRNA and short RNA/DNA chimeric elements (mRNA+RNA/DNA25B, mRNA+RNA/DNA60C). E–G) Evaluation of protein expression efficiency for ACE mRNA variants (mRNA/DNA25B, mRNA/DNA60C) encoding RFP compared to Ψ‐modified IVT mRNA and unmodified IVT mRNA. All mRNA samples were delivered to E) HeLa cells, F) HEK 293T, or G) Raw264.7 cells using LNP as the delivery method, and RFP expression was quantified 24 h post‐transfection. Statistical significance was determined using Student's *t*‐test (ns: not significant, *p < 0.05, **p < 0.01, and ***p < 0.001)

**Figure:**
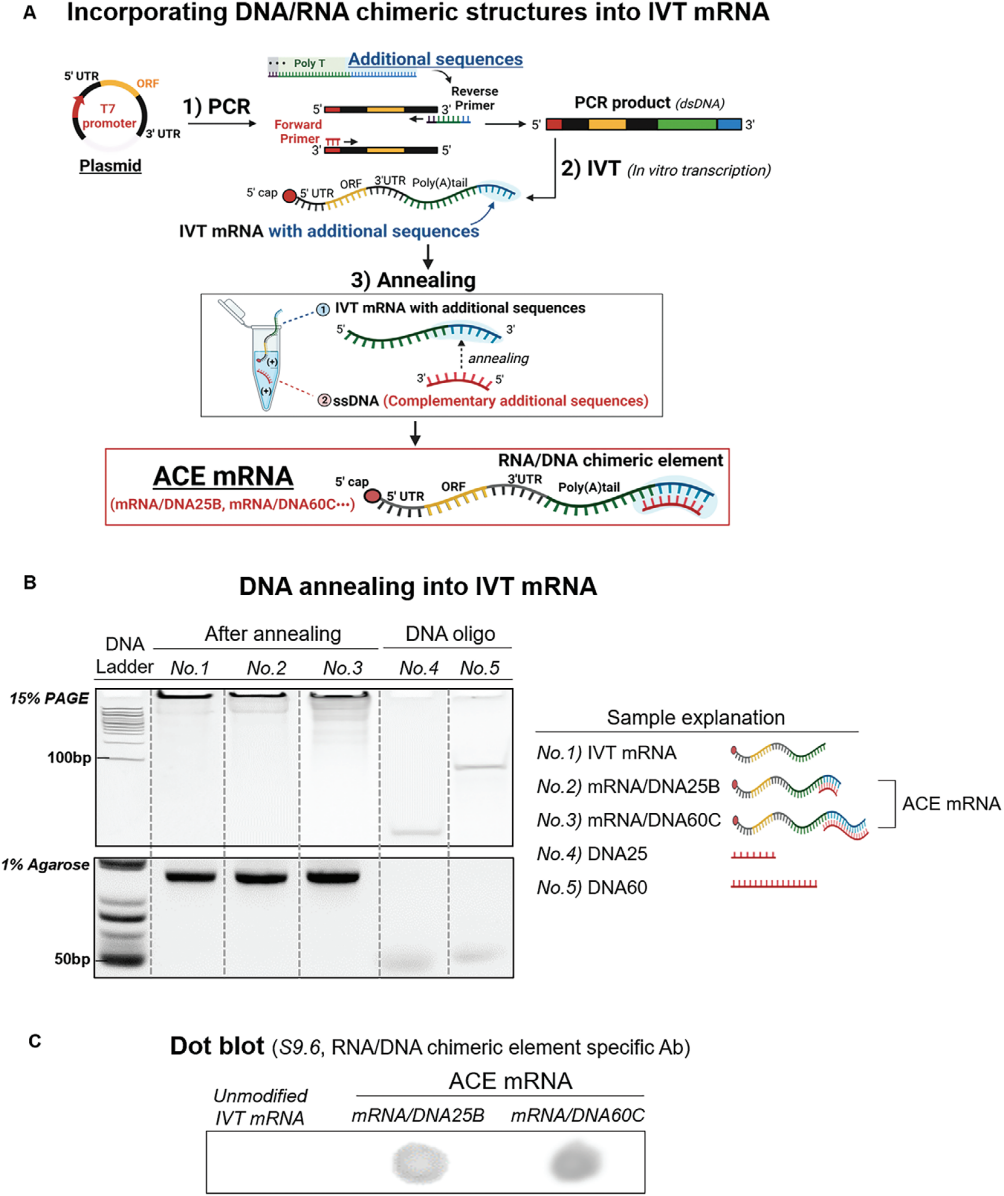

**Figure:**
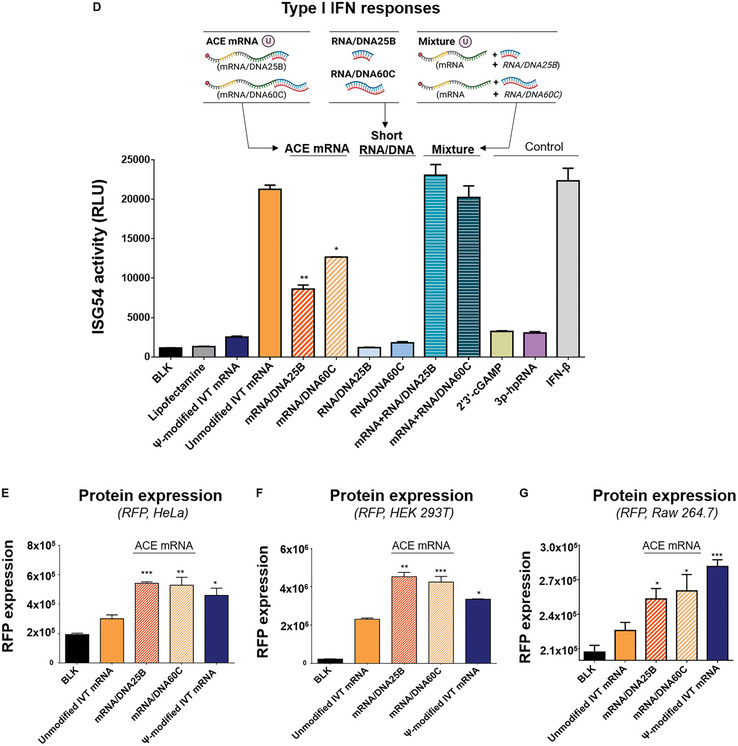

In the polyacrylamide gel electrophoresis assay, the DNA oligo bands (DNA25: complementary sequence to sequence B + 6 dT, DNA60: complementary sequence to sequence C + 11 dT) disappeared after the slow annealing process with IVT mRNA containing sequence B or C (Figure 2B). This result confirmed the successful introduction of RNA/DNA chimeric elements into the IVT mRNA. To assess the mRNA integrity of ACE mRNA, a 1% agarose gel electrophoresis assay was conducted, revealing that the bands corresponding to ACE mRNAs (mRNA/DNA25B and mRNA/DNA60C) were intact and similar to the band of IVT mRNA without RNA/DNA chimeric elements. To further confirm the successful incorporation RNA/DNA chimeric elements into the IVT mRNA, dot blot analysis was conducted using the S9.6 antibody, which specifically detects the RNA/DNA structures (Figure 2C). ACE mRNA showed a clear signal in the dot blot analysis, while no signal was observed with cellulose‐purified unmodified IVT mRNAs without RNA/DNA chimeric elements, indicating the successful incorporation of the RNA/DNA chimeric elements in the ACE mRNA.

### 2.3 ACE mRNA Reduced Type I IFN Responses of Unmodified IVT mRNA, Improving Protein Expression Efficiency

To verify the immunostimulatory effects of the prepared ACE mRNA, THP1‐Dual cells were treated with ACE mRNA in vitro using lipofectamine 2000. To further validate the distinct impact of the RNA/DNA chimeric elements present within ACE mRNAs, we also conducted additional assessments. These include samples with short RNA/DNA chimeric elements (RNA/DNA) that were not introduced into 3′ ends of IVT mRNA. These short RNA/DNA elements had exactly the same sequence and length as the RNA/DNA chimeric elements introduced into ACE mRNA (RNA/DNA25B for mRNA/DNA25B or RNA/DNA60C for mRNA/DNA60C). Additionally, we also verified the simple mixture of IVT mRNAs and the short RNA/DNA chimeric elements (mRNA + RNA/DNA25B or mRNA + RNA/DNA60C). All IVT mRNAs utilized in this assay were purified using cellulose purification steps for removing dsRNA by‐products. The comprehensive procedure for the preparation of ACE mRNA, including the step involving the removal of double‐stranded RNA (dsRNA), is provided in the supplementary information (Figure S2, Supporting Information). Subsequently, the levels of Lucia luciferase expression were measured as an indicator of ISG54 activity and type I IFN responses (Figure 2D). As a result, both variants of ACE mRNA (mRNA/25B and mRNA/DNA60C) demonstrated diminished ISG54 activity compared to the unmodified IVT mRNA, which suggested that the introduced RNA/DNA chimeric elements played a role in reducing the type I IFN responses of IVT mRNA. Notably, the immunomodulatory effects of the RNA/DNA chimeric elements were only observed when they were introduced at the 3′ ends of IVT mRNA as ACE mRNA. When RNA/DNA chimeric elements were treated alone or in a simple mixture with IVT mRNA, there was no noticeable change in the stimulation of ISG54 activities induced by IVT mRNA (Figure 2D). The noted immunomodulatory effects of ACE mRNA were also observed regardless of the capping agent used to prepare ACE mRNA. ACE mRNA prepared with CleanCap AG showed reduced ISG54 activity compared to the unmodified IVT mRNA prepared with CleanCap AG, which was similar to the results in Figure 2D obtained with ACE mRNA prepared with Anti Reverse Cap Analog (ARCA) (Figure S3, Supporting Information). To further confirm the immunoreduction effect of ACE mRNA in different cell lines, Raw‐Dual cells were treated with ACE mRNA (Figure S4, Supporting Information). While significant ISG54 activity was noted in cells treated with unmodified IVT mRNA, cells treated with ACE mRNA exhibited a substantial reduction in ISG54 activity.

To verify whether the reduced immunostimulatory effects of ACE mRNA affect the protein expression efficiency of IVT mRNA, both variants of ACE mRNA (mRNA/DNA25B or mRNA/DNA60C) were delivered to three different cell lines (HeLa, HEK 293T, and Raw 264.7) using lipid nanoparticles (LNP) as a delivery system. As the LNP system is widely used as a highly effective tool for the development of mRNA‐based therapeutics/vaccines, it was considered more appropriate to evaluate the protein expression efficiency of ACE mRNA within this system. In order to assess protein expression efficiencies in comparison to conventional IVT mRNAs, we also treated cells with IVT mRNA synthesized with unmodified nucleotides (unmodified IVT mRNA) and IVT mRNA incorporating Ψ modifications (Ψ‐modified IVT mRNA) using LNP. For this, one of the typical components of LNP (SM‐102 50%: DSPC 10%: Cholesterol 38.5%: DMG‐PEG2000 1.5%) was utilized in the formulation of various samples of IVT mRNAs including ACE mRNAs. In addition, the ORFs of all IVT mRNAs were encoded with a red fluorescent protein (RFP) to allow for a simple assessment of the protein expression efficiency. After the formulation of various IVT mRNA samples into LNP, the encapsulation efficiencies (EE), hydrodynamic sizes, and polydispersity (PDI) were verified. All variants of ACE mRNA were successfully loaded into the LNP, demonstrating RNA EE of over 90% (Figure S1A, Supporting Information). Furthermore, the LNP loaded with ACE mRNA exhibited a size of ≈100 nm and a polydispersity (PDI) of less than 0.2. These results were comparable to the LNP loaded with IVT mRNAs (unmodified IVT mRNA or Ψ‐modified IVT mRNA) without RNA/DNA chimeric elements (Figure S1B, Supporting Information). This suggests that the formulation of IVT mRNA into LNPs was not disturbed by the RNA/DNA chimeric elements incorporated into ACE mRNAs. After the LNP formulation, the type I IFN responses of ACE mRNA were examined in two different cell lines (THP1‐Dual cells and Raw‐Dual cells). The immunoreduction effect of ACE mRNA compared to unmodified IVT mRNA persisted after the LNP formulation, which was confirmed in both THP1‐Dual cells and Raw‐Dual cells (Figure S5, Supporting Information). To verify the protein expression efficiencies, the fluorescence intensities of RFP in cells were verified 24 h after the treatment with LNPs encapsulating various IVT mRNA samples. As a result, both variants of ACE mRNA (mRNA/DNA25B and mRNA/DNA60C) exhibited superior protein expression compared to unmodified IVT mRNA without RNA/DNA chimeric elements in all cell lines tested (HeLa, HEK 293T, and Raw264.7) (Figure 2E–G). Surprisingly, the enhanced protein expression of ACE mRNA was even better than the Ψ‐modified IVT mRNAs in HeLa and HEK 293T. In the mRNA‐mediated translational cascades, the activation of protein kinase R (PKR) leads to the inhibition of translation, a response that can be triggered by IVT mRNA. ACE mRNA showed diminished cellular PKR activity, suggesting that the influence of ACE mRNA on PKR‐related regulation of cellular translation could potentially enhance protein expression efficiency (Figure S6, Supporting Information). These results indicated that the inclusion of RNA/DNA chimeric elements at the 3′ ends of unmodified IVT mRNA resulted in an enhancement of protein expression, possibly due to a reduction in the stimulation of type I IFN.

To further confirm the potential for increased protein expression of ACE mRNA, additional ACE mRNA variants with different designs (mRNA/DNA21A mRNA/DNA65C) and additional ACE mRNA encoding three different proteins (RFP (ORF size: 678 nt), firefly luciferase (ORF size: 1653 nt), and *Streptococcus pyogenes* Cas9 (spCas9, ORF size: 4101 nt)) were synthesized. Subsequently, their protein expression efficiencies were verified in vitro (Figures S7,S8, Supporting Information). All additionally prepared ACE mRNA variants with different designs of RNA/DNA chimeric elements exhibited enhanced protein expression compared to their respective unmodified IVT mRNAs without RNA/DNA chimeric elements (Figure S7, Supporting Information). Overall, we prepared four ACE mRNA variants with different incorporated RNA/DNA chimeric element designs (mRNA/DNA21A, mRNA/DNA25B, mRNA/DNA60C, and mRNA/DNA65C), and they showed enhanced protein expression efficiency compared to the unmodified IVT mRNA (Figure S7, Supporting Information). Furthermore, ACE mRNA encoding different proteins with varying ORF sizes demonstrated enhanced protein expression compared to unmodified IVT mRNAs without RNA/DNA chimeric elements (Figure S8, Supporting Information). These results suggest that ACE mRNA can be applied to a broader range of RNA/DNA chimeric element designs and to encoded target proteins with different ORF sizes, exhibiting improved protein expression efficiency.

In the preparation of IVT mRNA, the plasmid DNA amplified in *Escherichia coli* (*E. Coli*) serves as a more suitable DNA template compared to the PCR product, as it eliminates the need for an additional PCR amplification step. To examine the practicality of ACE mRNA in terms of a straightforward production method, ACE mRNA was designed to be synthesized using plasmid DNA (Figure S9, Supporting Information). In this approach, the additional sequences should be integrated into the plasmid DNA, along with a poly(dA:dT) sequence. For this, it was important to consider that the homopolymer sequence of poly(dA:dT) can readily undergo recombination during *E. coli* bacterial amplification, resulting in a shortened length compared to the originally cloned sequence. The maximum length of poly(dA:dT) that can be introduced into plasmid DNA without encountering this shortening issue is known to be approximately 50 bp. In this regard, 50 bp of poly(dA:dT) and an additional sequence with an enzyme site were inserted downstream of the 3′UTR in plasmid DNA encoding firefly luciferase. The prepared plasmid DNA containing poly(dA:dT) and additional sequences was enzymatically cleaved for linearization, followed by IVT for the synthesis of IVT mRNA containing both a 50 nt poly(A) tail and additional sequences. The prepared IVT mRNA was then annealed to a DNA oligo with a complementary sequence to the additional sequence, resulting in the production of ACE mRNA using the plasmid DNA‐based method. As a control, unmodified IVT mRNA with a 50 nt poly(A) tail and without an RNA/DNA chimeric element was also prepared using the plasmid DNA‐based method. The ACE mRNA prepared with the plasmid‐based method showed enhanced protein expression compared to the unmodified IVT mRNA, consistent with the results obtained with the ACE mRNA produced by the PCR‐based method. This result confirms the significant potential of ACE mRNA for practical applications with its simplified production process.

### 2.4 ACE mRNA‐Mediated Reduction of Type I IFN Responses Is the Combined Result of the Restriction of RIG‐I and STING Activation and the Slight Induction of cGAS‐Mediated Immune Stimulation

**Figure 3:**
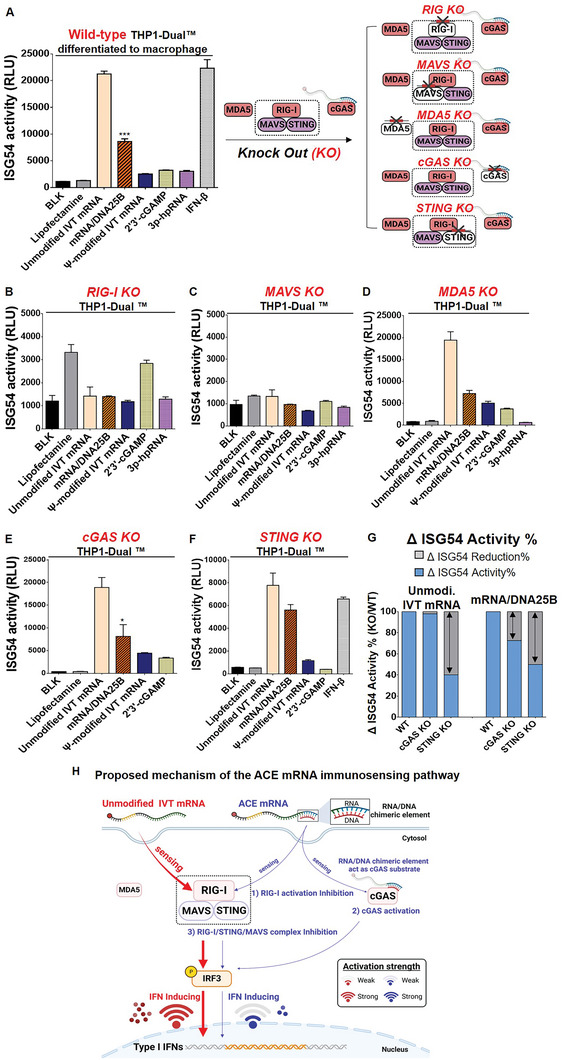
Type I IFN responses of ACE mRNA in wild‐type THP1‐Dual™ and various knock‐out (KO) cell lines of THP1‐Dual cells. To identify the specific PRRs responsible for decreasing the immune response of ACE mRNA, various knock‐out (KO) cell lines of THP1‐Dual cells were used. A) Wild‐type THP1‐Dual cells, B) RIG‐I KO THP1‐Dual cells, C) MAVS KO THP1‐Dual cells, D) MDA5 KO THP1‐Dual cells, E) cGAS KO THP1‐Dual cells, or F) STING KO THP1‐Dual cells were treated with various mRNA constructs, including ACE mRNA (unmodified IVT mRNA, ACE mRNA (mRNA/DNA25B), or Ψ‐modified IVT mRNAs) using Lipofectamine 2000. The type I IFN responses were assessed by quantifying Lucia luciferase expression in either wild‐type or various KO cell lines of THP1‐Dual cells. G) The extent of reduction in ISG54 activity on ACE mRNA in cGAS KO or STING KO THP1‐Dual cells compared to wild‐type THP1‐Dual cells H) Proposed mechanism of the ACE mRNA immunosensing pathway distinct from that of unmodified IVT mRNA. The overall capacity of ACE mRNA to reduce type I IFN responses is attributed to its potential to 1) inhibit the ATPase activity required for RIG‐I activation, thereby limiting RIG‐I‐mediated type I IFN responses, 2) engage in cGAS‐mediated immune activation, leading to a slight increase in type I IFN responses, and 3) restrict interactions between STING, RIG‐I, and MAVS, resulting in the inhibition of type I IFN responses. Statistical significance was ascertained using Student's *t*‐test (ns: not significant, *p < 0.05 and **p < 0.01).

To identify the specific PRRs responsible for decreasing the immune response of ACE mRNA, various knock‐out (KO) cell lines of THP1‐Dual cells were used. These cell lines were treated with various mRNA constructs (unmodified IVT mRNA or Ψ‐modified IVT mRNA) including ACE mRNA (mRNA/DNA25B) using lipofectamine 2000. The levels of Lucia luciferase expression were then measured to determine ISG54 activities, which indicate the activation of the IRF pathway and the production of type I interferon (IFN). In the wild‐type THP1‐Dual cell line, ACE mRNA (mRNA/DNA25B) showed a significant level of reduced ISG54 activities compared to unmodified IVT mRNA (**Figure** **3**A). When RIG‐I and mitochondrial antiviral signaling protein (MAVS) KO THP1‐Dual cell lines were treated, the ISG54 activities triggered by all mRNA constructs in the wild‐type THP1‐Dual cell line decreased to negligible levels (Figure 3B,C). Conversely, there was no noticeable change observed in the MDA5 KO THP1‐Dual cell line compared to the wild‐type THP1‐Dual cell line (Figure 3D). RIG‐I and MDA5 are cytoplasmic PRRs that recognize different types of nucleic acid structures based on their PAMPs and chemical modifications.^[^ ^36^ ^]^ Thus, it was hypothesized that RIG‐I and MDA5 recognize and sensitize the unmodified IVT mRNA, ACE mRNA (mRNA/DNA25B), or Ψ‐modified IVT mRNA in distinct ways. MAVS triggers immune responses that activate IRF3, IRF7, and NF‐kB by interacting with RIG‐I or MDA5, resulting in the stimulation of type I IFN production.^[^ ^37^, ^38^ ^]^ As MAVS acts as an adapter molecule downstream of both RIG‐I and MDA5, the absence of MAVS in the cell line diminishes all signals sensitized by RIG‐I and MDA5. Considering that the reduced immune responses were observed in the RIG‐I KO cell lines and not in the MDA5 KO cell lines, it can be concluded that the reduction of type I IFN responses observed in the MAVS KO cell lines was the result of the reduction of the RIG‐I mediated sensitization of the mRNA constructs. Overall, it was concluded that RIG‐I, in conjunction with the MAVS protein, rather than MDA5, plays a significant role in the type I IFN responses induced by all mRNA constructs including unmodified IVT mRNA, ACE mRNA (mRNA/DNA25B), and Ψ‐modified IVT mRNA. In addition, it was proposed that the observed ability of ACE mRNA to reduce type I IFN responses was predominantly due to the RIG‐I/MAVS pathway.

RIG‐I comprises two caspase recruitment domains, a C‐terminal regulatory domain, and a DExD/H‐box helicase domain responsible for its ATPase activity. In the context of RIG‐I‐mediated immune stimulation, two key factors play a crucial role in triggering immune stimulation: the binding of nucleic acid structures to RIG‐I and the ATPase activity of RIG‐I following the binding.^[^ ^33^ ^]^ The binding efficiency of the non‐self RNA to RIG‐I determines the initial steps of immune sensitization. Additionally, ATP hydrolysis is closely associated with the RIG‐I oligomerization, which represents a critical step in activating interconnected signaling pathways involving MAVS, which is necessary for the induction of type I IFN. It has been reported that the RNA/DNA chimeric nucleic acid structures may inhibit the ATPase activity of RIG‐I, thereby limiting RIG‐I activation and subsequent immune activation.^[^ ^33^ ^]^ Based on these reported results, it was thought that the RNA/DNA chimeric elements introduced at the 3′ ends of IVT mRNA might interfere with RIG‐I‐mediated immune activation by possibly restricting the ATPase activity.

Stimulator of interferon genes (STING) is a different category of cytoplasmic PRRs, known for their ability to recognize cyclic dinucleotides such as 2′3′‐cGAMP, which are produced by cyclic GMP‐AMP synthase (cGAS) using DNA as substrates.^[^ ^39^ ^]^ In this regard, the cGAS‐STING signaling pathway predominantly initiates innate immune responses by recognizing non‐self DNA, while RIG‐I or MDA5/MAVS distinguish non‐self RNA. In recent reports, it was suggested that the cGAS can also produce cyclic dinucleotides utilizing RNA/DNA chimeric elements as substrates, triggering immune signaling through STING.^[^ ^40^ ^]^ To examine whether RNA/DNA chimeric elements introduced to ACE mRNA influence the cGAS‐STING signaling pathway, cGAS or STING KO THP1‐Dual cells were examined. In both cGAS and STING KO THP1‐Dual cell lines, ACE mRNA showed reduced ISG54 activities compared to unmodified IVT mRNA (Figure 3E,F), indicating that the immunoreduction capacity of ACE mRNA is maintained in the absence of cGAS or STING. However, the levels of immunoreduction capability of ACE mRNA were slightly different in cGAS and STING KO cell lines. To directly compare the ISG54 activities of mRNA variants observed in two KO cell lines with those observed in wild‐type cell lines, relative ISG54 activities (%) were calculated (Figure 3G). In cGAS KO THP1‐Dual cells, the levels of ISG54 activity for ACE mRNA were slightly decreased compared to that detected in wild‐type cell lines (27.37%), while no notable change was detected for unmodified IVT mRNA (2.04%) (Figure 3G). This means that the ability of ACE mRNA to reduce type I IFN responses was enhanced in the absence of cGAS. In other words, the immunoreduction capacity of ACE mRNA can be enhanced in the absence of cGAS. As a possible reason, it was thought that the RNA/DNA chimeric elements introduced to ACE mRNA can partially serve as a substrate for cGAS and thus have the potential to be partially involved in the activation of type I IFN responses. In this sense, it was suggested that the ability of ACE mRNA in the reduction of type I IFN responses was partially abolished by cGAS‐related immune activation. Meanwhile, when STING KO THP1‐Dual cells were examined, a significant decrease in ISG54 activity was observed in both unmodified IVT mRNA and ACE mRNA compared to the wild‐type THP1‐Dual cells (Figure 3G). This indicates that STING plays a partial role in IVT mRNA‐mediated immune activation. Furthermore, if the level of reduction in ISG54 activity on ACE mRNA in STING KO THP1‐Dual cells compared to wild‐type THP1‐Dual cells is similar to that on unmodified IVT mRNA, then it can be concluded that there is no unique mechanism of STING‐related immune response to ACE mRNA apart from what is observed with unmodified IVT mRNA. However, the results showed that ACE mRNA and unmodified IVT mRNA exhibited different levels of reduction in ISG54 activity (Figure 3G). While a 59.74% reduction in ISG54 activity was observed for unmodified IVT mRNA, a 49.95% reduction was detected for ACE mRNA, which is a slightly smaller reduction compared to unmodified IVT mRNA. The difference in the reduction levels observed in ACE mRNA compared to unmodified IVT mRNA suggests one of the possible mechanisms of the immunoreduction capacity of ACE mRNA, which is associated with STING. A smaller level of reduction suggests the possible mechanism of ACE mRNA in inhibiting STING‐related immune responses of unmodified IVT mRNA. Several studies have reported that STING interacts with RIG‐I and MAVS, playing a role in the transmission of RIG‐I signaling.^[^ ^41^, ^42^ ^]^ This interaction produces a complex involving STING, RIG‐I, and MAVS, which ultimately triggers the production of type I IFN, independently of cGAS. In this context, it is likely that ACE mRNA might interfere with the interactions among STING, RIG‐I, and MAVS, potentially hindering the complex formation and reducing type I IFN activation. If the ability of ACE mRNA to downregulate type I IFN responses was solely related to the STING‐related immune activation, the level of ISG54 activity for ACE mRNA in STING KO THP1‐Dual cells would return back to the level of unmodified IVT mRNA. Nevertheless, the results showing that ACE mRNA in STING KO THP1‐Dual cells still exhibited reduced levels of ISG54 activity compared to unmodified IVT mRNA suggest that other factors independent of STING, such as the aforementioned RIG‐I activation, cooperate with STING‐associated immune activation to influence the immunoreduction capacity of ACE mRNA.

Taking these results into consideration, it was concluded that ACE mRNA restricts IVT mRNA‐mediated RIG‐I and STING activation, leading to a reduction in type I IFN responses (Figure 3H). The overall ability of ACE mRNA to reduce type I IFN is based on its potential to 1) inhibit the ATPase activity required for RIG‐I activation, limiting RIG‐I mediated type I IFN responses, 2) participate in cGAS mediated immune activation, slightly increasing type I IFN responses, 3) limit interactions between STING, RIG‐I and MAVS, inhibiting type I IFN responses (Figure 3H).

### 2.5 ACE mRNA Significantly Enhanced the Efficiency of Therapeutic Protein Expression (hEPO) of Unmodified IVT mRNA Without Chemical Modification In Vivo

**Figure 4:**
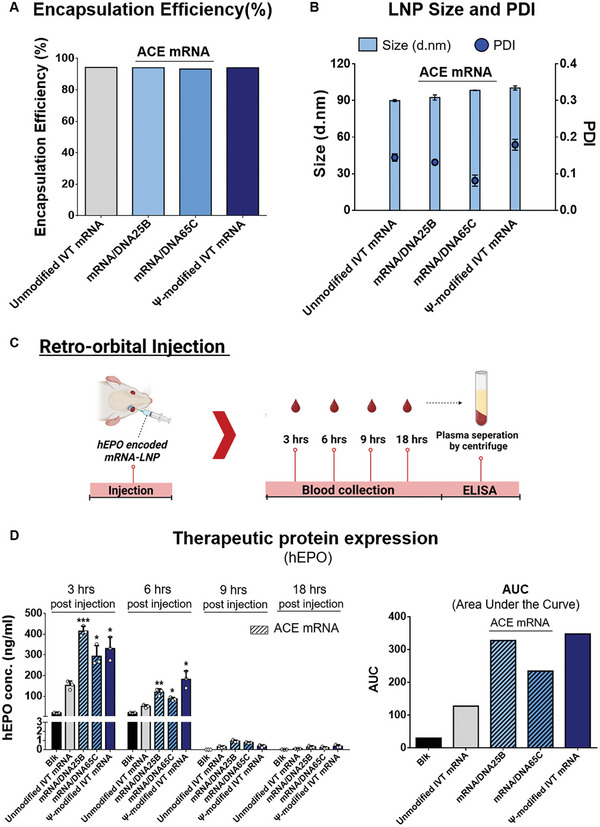
Therapeutic protein expression (hEPO) of ACE mRNA in vivo. The ACE mRNA variants (mRNA/DNA25B, mRNA/DNA65C) and IVT mRNAs (unmodified IVT mRNA and Ψ‐modified IVT mRNA encoding hEPO) were formulated into LNP to verify the efficiency of therapeutic protein expression in vivo. A) The encapsulation efficiency (%) of all mRNA constructs was measured by a ribogreen assay. B) The hydrodynamic sizes and PDI of the formulated LNPs were measured using dynamic light scattering (DLS). C) Schematic representation illustrating the in vivo administration of LNP encapsulating various mRNA samples encoding hEPO. A total of 2 micrograms of each mRNA variant were administered retro‐orbitally (*n* = 3). Blood collection was performed at 3, 6, 9, and 18 h post‐injection using a Lancet, and the collected blood was immediately mixed with 3.8% sodium citrate buffer to prevent coagulation. Subsequently, the collected blood was subjected to centrifugation for plasma separation. D) The levels of produced hEPO in the collected plasma samples were examined using an enzyme‐linked immunosorbent assay (ELISA). In addition, the total protein expression was verified by calculating the area under the curve (AUC) using detected hEPO concentration in serum samples collected at 4 time points (3, 6, 9, and 18 h post‐injection). Statistical significance was determined through a Student's *t*‐test (ns: not significant, *p < 0.05, **p < 0.01, and ***p < 0.001)

To evaluate the practical clinical applications of ACE mRNA for therapeutic protein expression, the coding sequence of human erythropoietin (hEPO) was incorporated into the ORF sequence of ACE mRNA. Two variants of ACE mRNA, mRNA/DNA25B, and mRNA/DNA65C, with 25 and 65 bp of RNA/DNA chimeric element introduced at the 3′ ends of unmodified IVT mRNA, respectively, were used. The hEPO protein expression levels of ACE mRNA (mRNA/DNA25B and mRNA/DNA65C) were compared with the unmodified IVT mRNA and Ψ‐modified IVT mRNA, all of which encode hEPO. All variant mRNA constructs were formulated into LNP (SM‐102 50%: DSPC 10%: Cholesterol 38.5%: DMG‐PEG2000 1.5%), followed by measurement of EE, hydrodynamic size, and PDI (**Figure** **4**A,B). The EE of all mRNA constructs was measured to be above 90%, indicating sufficient loading efficiency, regardless of whether RNA/DNA chimeric elements or Ψ chemical modification were introduced into the IVT mRNA. In addition, the size and PDI of the formulated LNP were consistently below 100 nm and 0.2, respectively, for all mRNA constructs (Figure 4B). This confirmed that no matter whether the introduction of RNA/DNA chimeric element into the IVT mRNA, it can be successfully loaded into the LNP with high quality, which is consistent with the above results obtained by using mRNA variants encoding RFP.

The LNPs formulated with mRNA variants (unmodified IVT mRNA, ACE mRNA (mRNA/DNA25B and mRNA/DNA65C), Ψ‐modified IVT mRNA) encoding hEPO were injected into mice by retro‐orbital injection at a dose of 0.1 mpk (mg kg^−1^), and serum samples were collected at different time points (3, 6, 9, and 18 h) after the injection (Figure 4C). The concentrations of produced hEPO were measured in the collected plasma samples by using enzyme‐linked immunosorbent assay (ELISA). At 3 h post‐injection, all ACE mRNA variants (mRNA/DNA25B and mRNA/DNA65C) exhibited superior hEPO expression compared to unmodified IVT mRNA. The enhanced protein expression efficiencies of ACE mRNA variants were comparable to that of Ψ‐modified IVT mRNA (Figure 4D). These results demonstrate that ACE mRNA can notably improve the therapeutic protein expression efficacy of unmodified IVT mRNA without the need for chemical modification techniques, confirming the capability of ACE mRNA in practical clinical applications as a technological alternative to chemical modification. In addition, among two ACE mRNA variants, mRNA/DNA25B having 25 bp of RNA/DNA chimeric elements exhibited more efficient hEPO expression compared to mRNA/DNA65C having 65 bp of RNA/DNA chimeric elements. These results showed that the overall length of the introduced RNA/DNA chimeric element may affect the protein expression efficiency of ACE mRNA. This suggests that there is still potential for further optimization of the total length of the RNA/DNA chimeric elements introduced to 3′ ends of IVT mRNA to increase the protein expression efficiency of unmodified IVT mRNA. In the results obtained with serum samples collected at 6 h post‐injection, both ACE mRNAs showed enhanced hEPO expression superior to unmodified IVT mRNA consistently with results obtained at 3 h post‐injection. It was noteworthy that the degree of increased protein expression efficiency observed with ACE mRNA at 3 h post‐injection was somewhat diminished at 6 h post‐injection. To further examine this observation, the immunoreduction effects of ACE mRNA were systematically examined over time using THP1‐Dual cells (Figure S10, Supporting Information). The type I IFN reduction effect of ACE mRNA detected at 6 h post‐treatment was decreased at 24 h post‐treatment. It was thought that this decrease was due to the removal of RNA/DNA chimeric elements introduced to ACE mRNA by intracellular RNase H. To verify the total protein expression efficiency over an extended period, the area under the curves (AUC) was calculated using the detected concentrations of hEPO in serum samples collected at four different time points (3, 6, 9, and 18 h after the injection) (Figure 4D). The calculated AUC, representing the total protein expression efficiency, of the ACE mRNA was better than that of the unmodified IVT mRNA and comparable with that of the Ψ‐modified IVT mRNA. These results strongly suggested the great potential of ACE mRNA as an innovative IVT mRNA platform for enhanced therapeutic protein production.

### 2.6 RNA/DNA Chimeric Element Incorporated into Ψ‐Modified IVT mRNA Reversely Affects Immune Modulation, Increasing Type I IFN Responses

**Figure 5:**
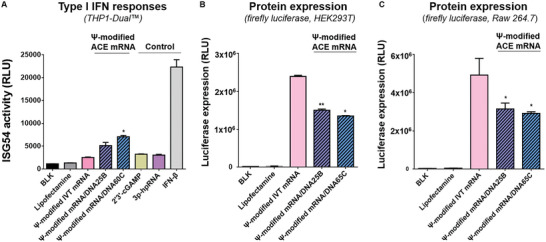
Type I IFN response and protein expression of Ψ‐modified IVT mRNA incorporating RNA/DNA chimeric element (Ψ‐modified ACE mRNA). A) THP1‐Dual cells were treated with Ψ‐modified IVT mRNA and Ψ‐modified ACE mRNA (mRNA/DNA25B, mRNA/DNA65C) to measure ISG54 activity. B) HEK 293T cells and Raw 264.7 cells were transfected with Ψ‐modified IVT mRNA or Ψ‐modified ACE mRNA variants (mRNA/DNA25B, mRNA/DNA65C) encoding firefly luciferase, and the efficiency of protein expression was determined by quantifying luciferase expression levels.

We have successfully confirmed that ACE mRNA, based on unmodified IVT mRNA with RNA/DNA chimeric elements incorporated at its 3′ ends, contributes to the reduction of type I IFN responses and consequently improves the efficiency of therapeutic protein expression. Following this discovery, we became curious about the consequences of introducing RNA/DNA chimeric elements to chemically modified IVT mRNA. To investigate the effect of RNA/DNA chimeric elements on immunomodulation when introduced into Ψ‐modified IVT mRNA, we prepared Ψ‐modified IVT mRNA containing RNA/DNA chimeric elements at its 3′ ends (Ψ‐modified ACE mRNA). The sequences and lengths of the RNA/DNA chimeric elements introduced into the Ψ‐modified ACE mRNA were exactly the same as those used in the ACE mRNA prepared with the unmodified IVT mRNA described above. Subsequently, the prepared Ψ‐modified ACE mRNAs were treated with wild‐type THP1‐Dual cells for further analysis. Based on the previous results of ACE mRNA prepared with unmodified IVT mRNA, its potential mechanism for immnoreduction can be attributed to the combined effects of 2 downregulation mechanisms involving reduction of RIG‐I activity and restricting STING‐related, cGAS‐independent immune responses, and 1 upregulation mechanism slightly activating cGAS‐related immune responses. Dissecting these hypotheses, 2 downregulation mechanisms are related to the immunostimulatory effect of unmodified IVT mRNA, where the role of the RNA/DNA chimeric element is to limit the immune stimulation effect of unmodified IVT mRNA, and 1 upregulation mechanism is related to the RNA/DNA chimeric element potentially acting as a substrate for the cGAS‐mediated immunostimulatory pathway independently of unmodified IVT mRNA. This implies that the ability of RNA/DNA chimeric elements to restrict the immunostimulatory effect of unmodified IVT mRNA may be diminished by the involvement of the cGAS‐mediated immunostimulatory pathway. Since the Ψ‐modified IVT mRNA showed negligible immune activation linked to RIG‐I and STING‐related, cGAS‐independent immune pathway, it is plausible that the 2 downregulation mechanisms shown in ACE mRNA prepared with unmodified IVT mRNA may not exert an influence on the Ψ‐modified ACE mRNA. Instead, the 1 upregulation mechanism, which triggers cGAS‐related immune responses, might play a predominant role in immune responses induced by Ψ‐modified ACE mRNA, consequently leading to increased immune reactions. As hypothesized, both variants of Ψ‐modified ACE mRNA (Ψ‐modified mRNA/DNA25B and Ψ‐modified mRNA/DNA60C) showed increased ISG54 activities, representing the induction of type I IFN responses, in comparison to the Ψ‐modified IVT mRNA not incorporating RNA/DNA chimeric elements (**Figure** **5**A). This result indicates that immunosilent Ψ‐modified IVT mRNA can be adjusted toward upregulation in immune responses by introducing RNA/DNA chimeric elements at their 3′ ends, by acting as a substrate of cGAS. Among the two variants of Ψ‐modified ACE mRNA, Ψ‐modified mRNA/DNA60C induced more type I IFN responses than Ψ‐modified mRNA/DNA25B. This result suggests that the longer RNA/DNA chimeric elements (60 bp) introduced into Ψ‐modified mRNA/DNA60C can serve as a more effective substrate for cGAS than the 25 bp RNA/DNA chimeric elements introduced into Ψ‐modified mRNA/DNA25B. It's worth noting that when using unmodified nucleotides, mRNA/DNA60C exhibited fewer immunoreduction effects compared to mRNA/DNA25B (as shown in Figure 2D). This can likely be attributed to the relatively more efficient activation of cGAS by the longer RNA/DNA chimeric elements (60 bp) introduced into unmodified mRNA/DNA60C as compared to the 25 bp RNA/DNA chimeric elements introduced into unmodified mRNA/DNA25B, which more diminished the 2 downregulation effects associated with RIG‐I and STING. To examine the protein expression efficiency of Ψ‐modified ACE mRNA, two variants of Ψ‐modified ACE mRNA (Ψ‐modified mRNA/DNA25B and Ψ‐modified mRNA/DNA65C) encoding firefly luciferase were transfected into two cell lines (HEK 293T and Raw 264.7), followed by measuring luciferase activity 24 h post‐transfection. As a result, two variants of ACE mRNA exhibited reduced protein expression compared to the Ψ‐modified IVT mRNA in both cell lines, likely to be reasoned by the increased immune responses observed in Ψ‐modified ACE mRNA (Figure 5B). In the context of vaccine development, which requires appropriate levels of both immune stimulation and translation, although the Ψ‐modified ACE mRNA showed reduced protein expression, the increased immune responses could be beneficial in the induction of protective immunity, resulting in improved vaccine efficacy.

## 3 Conclusion

In the context of nucleic acid structures, a range of structures has been designed and refined to modulate immunogenicity. Examples of such structures include CpG^[^ ^43^, ^44^ ^]^ and poly(I:C),^[^ ^45^, ^46^ ^]^ which have been specifically designed to be readily recognized by PRRs. This recognition then triggers innate immune responses, acting as adjuvants that ultimately improve vaccine efficacy, particularly for immunosilent subunit vaccines. Up until now, the design of nucleic acid structures for immunomodulation has largely focused on the induction of immune responses. Given the critical need to reduce the excessive immunostimulatory effects of IVT mRNA to achieve sufficient clinical efficacy, it is clear that the focus should be on reducing immune stimulation rather than inducing it. In this respect, there is a significant gap between the accumulated technical knowledge of nucleic acid structures in PRR recognition and their application in the field of mRNA therapeutics/vaccines. Recent reports have suggested that certain nucleic acid structures might possess the capacity to reduce immune activation by bypassing PRR‐mediated sensitization of non‐self RNAs.^[^ ^25^, ^31^, ^32^ ^]^ This implies that such nucleic acid structures could hold substantial potential in the clinical applications of IVT mRNA by contributing to the attenuation of immune activation.

In the case of IVT mRNA, modifying the nucleic acid structures can be achieved by sequence changes that affect the secondary or tertiary structures of RNA. However, the options for changing the sequence of IVT mRNA are limited due to the requirement of essential mRNA elements for translation, such as the 5′ cap, 5′ UTR, ORF, 3′ UTR, and poly(A) tail. Precise integration of these essential mRNA elements with an optimized sequence is required to ensure proper mRNA translation. The ORF sequence is determined by the protein it encodes, and modifying the optimal sequence of 5′ UTR and 3′ UTR may ultimately reduce protein expression efficiency. Furthermore, the incorporation of diverse non‐canonical nucleic acid structures into IVT mRNA is also challenging since maintaining the ssRNA form is essential for optimal mRNA function and adequate translation. As a strategy to incorporate additional elements capable of modulating immune responses without losing the function of IVT mRNA, we suggested the ACE mRNA that incorporates RNA/DNA chimeric elements at the 3′ ends of IVT mRNAs. The ACE mRNA includes two segments, one is based on the conventional IVT mRNA and the other is Additional Chimeric Elements, specifically RNA/DNA chimeric element that has the potential to modulation of immunogenicity of IVT mRNA. It was hypothesized that the introduced chimeric structure could reduce the immunogenicity of unmodified IVT mRNA by restricting PRRs‐mediated immune stimulation. In addition, it was anticipated that the introduced RNA/DNA chimeric elements would not interfere with mRNA translation because the chimeric elements can be removed from IVT mRNAs once they are delivered into cells, because of the presence of intracellular RNase H, which possesses the capacity to eliminate RNA/DNA chimeric elements.

The results presented in this study demonstrate the effectiveness of ACE mRNA in reducing type I IFN responses and enhancing protein expression efficiency. The IVT mRNA‐mediated induction of type I IFN responses is closely related to the dsRNA by‐products that are generated during the synthesis of IVT mRNA. Cellulose purification, a commonly used method, effectively removes dsRNA by‐products and eventually improves protein expression efficiency of unmodified IVT mRNA but is not sufficient to completely reduce type I IFN responses. Therefore, there is much room for improving the protein expression efficiency of unmodified IVT mRNA by reducing the induction of type I IFN. ACE mRNA prepared by introducing RNA/DNA chimeric elements into the 3′ends of unmodified IVT mRNA, the unmodified IVT mRNA‐mediated immune responses were significantly reduced. Additionally, the introduced chimeric elements did not interfere with the translation functions of IVT mRNA, achieving improved protein expression efficiency.

Further investigation into the mechanisms of ACE mRNA's immunomodulatory effects revealed that it reduced type I IFN responses primarily through the RIG‐I/MAVS pathway, rather than MDA5/MAVS. The RNA/DNA chimeric elements were proposed to inhibit RIG‐I's ATPase activity, thereby limiting RIG‐I activation and downstream immune signaling.^[^ ^33^ ^]^ Furthermore, the chimeric elements potentially served as substrates for cGAS, leading to a slight increase in type I IFN responses. It was also suggested that ACE mRNA interferes with interactions among STING, RIG‐I, and MAVS, which may contribute to reducing type I IFN activation. Overall, it was concluded that the observed immunoreduction effects of ACE mRNA are based on the end results of 2 immune downregulation mechanisms (limiting RIG‐I mediated type I IFN responses and limiting interactions between STING, RIG‐I, and MAVS) and 1 immune upregulation mechanism (cGAS mediated immune activation).

To assess the clinical relevance of ACE mRNA, we evaluated its impact on therapeutic protein expression (hEPO) in vivo. Two variants of ACE mRNA (mRNA/DNA25B and mRNA/DNA65C) showed significantly enhanced hEPO expression compared to unmodified IVT mRNA without chemical modification, and the protein expression efficiency was comparable to Ψ‐modified IVT mRNA. These results suggest that ACE mRNA has great potential as an alternative platform for improving therapeutic protein production without the need for costly chemically modified nucleotides.

Finally, we also investigated the effects of introducing RNA/DNA chimeric elements into Ψ‐modified IVT mRNA. It was hypothesized that the immune‐modulating effects of ACE mRNA based on unmodified IVT mRNA might be reversed when incorporated into Ψ‐modified IVT mRNA. However, it was noteworthy that Ψ‐modified IVT mRNA exhibits negligible immune activation linked to RIG‐I and STING‐related, cGAS‐independent immune pathways. Therefore, the immunoreduction capability of RNA/DNA chimeric elements observed in ACE mRNA prepared with unmodified nucleotides might be not workable when the chimeric elements are introduced into immunosilent Ψ‐modified IVT mRNA. Instead, the 1 immune upregulation mechanism, which originated from introduced RNA/DNA chimeric elements serving as substrates for cGAS, would be a predominant mechanism determining the final effect of Ψ‐modified ACE mRNA in immune responses. Indeed, Ψ‐modified ACE mRNA showed increased immune responses compared to Ψ‐modified IVT mRNA, thereby exhibiting decreased protein expression efficiency.

In conclusion, we developed ACE mRNA as a novel approach for modulating immune responses and controlling the protein expression efficiency of IVT mRNAs. By incorporating RNA/DNA chimeric elements into unmodified IVT mRNA, ACE mRNA effectively reduces type I IFN responses and exhibits enhanced protein expression. In addition, by incorporating RNA/DNA chimeric elements into Ψ‐modified IVT mRNA, ACE mRNA exhibited slight induction of type I IFN as compared to immunosilent Ψ‐modified IVT mRNA. Even though the Ψ‐modified ACE mRNA showed reduced protein expression, the induction of immune responses might be beneficial in terms of achieving vaccine efficacy that requires some level of immune stimulation. This finding strongly suggests the great potential of ACE mRNA in broadening the immunobalance of IVT mRNAs between immunosilent Ψ‐modified IVT mRNA and immunostimulatory unmodified IVT mRNA. By introducing RNA/DNA chimeric elements into IVT mRNAs produced with different chemically modified nucleotides, we can fine‐tune the immune response of IVT mRNA, providing various options for finding the right balance between inversely related immune stimulation and translation. Looking forward, further optimization and investigation are needed to fully understand the impact of RNA/DNA chimeric elements on chemically modified IVT mRNA other than Ψ‐modified IVT mRNA and their interaction with various immune pathways.

## 4 Experimental Section

### PCR

To synthesize IVT mRNAs, PCR products served as the DNA template for the in vitro transcription (IVT) reaction. The PCR products were produced using the TOPsimple PCR DryMIX‐Forte kit (Enzynomics, South Korea). To introduce additional sequences (sequence B or C) downstream of the poly(A) tail in the IVT mRNA, the reverse primers containing 120 nucleotides (nt) of poly(T) sequences, along with the designed additional sequences (sequence B: 5′‐AAGCAAGCTGACCCTGAAGTT‐3′ for mRNA/DNA25B, sequence C: 5′‐AACAGAGCGATGGGTATGTGGAGGGTTGTTAGATGGGAGATGGGTGGGGGC‐3′ for mRNA/DNA60C, or sequence C′: 5′‐AATGGGGGAGAGACAGAGCGATGGGTATGTGGAGGGTTGTTAGATGGGAGATGGGTGGGGGC‐3′ for mRNA/DNA65C) were utilized in the PCR process.

The prepared PCR products were analyzed by 1% agarose gel electrophoresis and subsequently purified using an AccuPrep PCR/Gel Purification Kit according to the manufacturer's instructions.

### IVT mRNA Synthesis

IVT mRNAs with or without additional sequences were synthesized by in vitro transcription (IVT) using EZ MEGA T7 Transcription kit (Enzynomics, South Korea) following the manufacturer's provided guidelines. During IVT reaction, ARCA (Anti‐reverse cap analog, 3′‐O‐Me‐7mG(5′)ppp(5′)G, NEB, USA) cap or CleanCap AG (Trilink, USA) was incorporated into IVT mRNAs alongside various chemically modified nucleotides (pseudouridine‐5′‐triphosphate (Ψ, TriLink Biotechnologies, USA) for Ψ‐modified IVT mRNA, 5‐methylcytidine‐5′‐triphosphate(5mC, TriLink Biotechnologies, USA) for 5mC‐modified IVT mRNA, or double modification involving both Ψ and 5mC for Ψ,5mC‐modified IVT mRNA). The synthesized IVT mRNAs with their respective chemical modifications were purified using the RNeasy mini kit (Qiagen, USA). The purified IVT mRNAs were either used directly or underwent additional purification through the cellulose purification method, and their quality was assessed using an Advanced analytical fragment analyzer (Agilent, USA) in accordance with the manufacturer's guidelines.

### Cellulose Purification

The purification of the cellulose was carried out according to a previously described method.^[^ ^35^ ^]^ Cellulose purification was used to remove the dsRNA by‐products from unmodified or chemically (Ψ‐, 5mC‐, or Ψ, 5mC) modified IVT mRNA. The cellulose fibers (Sigma–Aldrich, USA) were suspended in chromatography buffer (10 mm HEPES (pH 7.2), 0.1 mm EDTA, 125 mm NaCl, and 16% (v/v) ethanol) to create a cellulose slurry. This slurry was then subjected to centrifugation for 1 min at 14 000 RCF in a spin column, with this process being repeated twice as a prewashing step. The IVT mRNA, which was dissolved in 500 µL chromatography buffer, was placed into the prewashed cellulose fiber spin column for 30 min and then centrifuged at 14 000 RCF within the spin column for 1 min. This procedure effectively separated the dsRNA contaminants from the ssRNA and was repeated twice for thorough purification. Finally, the chromatography buffer was removed from the separated ssRNA via filtration using an Amicon Ultra system (Merck Millipore, USA), and the IVT mRNA dissolving buffer was changed to sterile‐filtered water treated with diethyl pyrocarbonate (DEPC water).

### Preparation of ACE mRNA

IVT mRNA constructs were prepared, each encoding different proteins such as firefly luciferase (Luciferase), red fluorescent protein (RFP), human erythropoietin (hEPO), and *Streptococcus pyogenes* Cas9 (spCas9). These mRNA structures contained 120‐nt poly(A) tails at their 3′‐ends. To introduce RNA/DNA chimeric elements into the ACE mRNA structure, the IVT mRNA, which had additional sequences at its 3′ ends, underwent a gradual annealing process with DNA oligomers (21‐nt DNA oligo for mRNA/DNA21A: 5′‐ GACACTAGTGGTCTCTTTTTT‐3′, 25‐nt DNA oligo for mRNA/DNA25B: 5′‐GCAAGCTGACCCTGAAGTTTTTTTT‐3′, 60‐nt DNA oligo for mRNA/DNA60C: 5′‐CAGAGCGATGGGTATGTGGAGGGTTGTTAGATGGGAGATGGGTGGGGGCTTTTTTTTTTT‐3′, 65‐nt DNA oligo for mRNA/DNA65C: 5′‐TGGGGGAGAGACAGAGCGATGGGTATGTGGAGGGTTGTTAGATGGGAGATGGGTGGGGGCTTTTT‐3′). This slow annealing process involved a stepwise decrease in temperature from 75 to 30 °C, with each 5 °C reduction lasting for 20 s, followed by rapid cooling to 4 °C. The annealing was carried out in a 1X phosphate‐buffered saline (PBS) solution, and the molecular ratio of IVT mRNA to DNA oligo in the annealing mixture was 1:0.5. To confirm the successful annealing, IVT mRNA, ACE mRNA, and DNA oligos were subjected to analysis by 15% polyacrylamide gel electrophoresis (PAGE) and 1% agarose gel electrophoresis both before and after annealing. The presence of DNA oligo bands and their disappearance after incorporation into ACE mRNA were confirmed using the Chemi‐Doc gel imaging system (Bio‐Rad, USA) and the Image Lab software.

### Dot Blot

The dot blot assay was performed according to a previously described method.^[^ ^35^ ^]^ Before and after cellulose purification, unmodified IVT mRNA and chemically modified IVT mRNA (Ψ‐, 5mC‐, or Ψ, 5mC) were diluted in DEPC water to a concentration of 250 µg/5 µL. These diluted solutions were subsequently applied onto a positively charged nylon transfer membrane (Nytran SPC, Sigma Aldrich, USA). To apply the solutions more easily onto the membrane, the membrane was securely fixed in place using a silicone mask. Following the loading of all IVT mRNA samples onto the membrane, dsRNA was identified using a primary antibody known as J2 (at a dilution of 1:5000, Scicons, Hungary). The J2 primary antibody was incubated overnight, after which a secondary antibody (at a dilution of 1:10 000, goat anti‐mouse IgG(H+L)‐HRP, GenDepot, USA) was applied in a solution of 1% skim milk. The dot blot was captured using the Chemi‐Doc gel imaging system. To quantify the amount of dsRNA, both before and after cellulose purification, the intensity of the dots detected in the dot blot analysis was calculated using the ImageLab software, and the results were presented by adjusting the band volume. For the purpose of confirming the presence of RNA/DNA chimeric elements, an S9.6 primary antibody was used. The unmodified IVT mRNA and ACE mRNA structures (mRNA/DNA25B and mRNA/DNA60C) were diluted in DEPC water to a concentration of 1 µg/5 µL and then loaded onto a positively charged nylon transfer membrane (Nytran SPC, Sigma Aldrich, USA), which was secured using a silicone mask. Following the loading of all mRNAs, the RNA/DNA chimeric elements were detected using the S9.6 primary antibody (at a dilution of 1:20 000, Kerafast, USA). This primary antibody was incubated overnight, and subsequently, a secondary antibody (at a dilution of 1:10000, goat anti‐mouse IgG(H+L)‐HRP, GenDepot, USA) was applied in a solution containing 1% skim milk. Dot blots were captured using Chemi‐Doc gel imaging systems.

### LNP Formulation and Characterization

The formulations of LNP were prepared by the optimized process of a previously described method.^[^ ^47^ ^]^ The IVT mRNAs (unmodified IVT mRNA, Ψ‐modified IVT mRNA) as well as ACE mRNAs (mRNA/DNA25B, mRNA/DNA60C, or mRNA/DNA65C) encoding either RFP or EPO were encapsulated into LNP. An ionizable lipid (SM‐102, MCE, USA), 1,2‐distearoyl‐sn‐glycero‐3‐phosphocholine (DSPC, Avanti polar lipids, USA), cholesterol (Avanti polar lipids, USA), and 1,2‐dimyristoyl‐rac‐glycero‐3‐methoxypolyethylene glycol‐2000 (DMG‐PEG 2000, Avanti polar lipids, USA) were mixed in ethanol in a specific molar ratio of 50:10:38.5:1.5. The IVT mRNAs or ACE mRNAs were diluted in an aqueous buffer (25 mm sodium acetate buffer (pH 5.0)) and rapidly mixed with an ethanol solution containing lipid mixture at a volume ratio of 3:1 (aqueous:ethanol) using a Nanoassemblr Ignite (Precision Nanosystems, Canada). The resulting LNPs were subjected to overnight dialysis against 20 mm Tris buffer (with a pH of 7.4) containing 8% sucrose, employing Slide‐A‐Lyzer Dialysis cassettes with a molecular weight cutoff of 3500 (Thermo Scientific, USA). The hydrodynamic sizes and PDI of the formulated LNPs were determined via dynamic light scattering (DLS) using the Zeta sizer (Malvern, UK). In addition, the encapsulation efficiency (EE, %) of RNA was confirmed by measuring the RNA concentration before and after treatment of LNPs with Triton‐X 100 using the Quant‐iT RiboGreen RNA assay kit (Invitrogen, USA).

### Cell Lines

HeLa cells (human cervical cancer cell line, Korea Cell Line Bank, South Korea) and HEK 293T cells (human embryonic kidney‐293T, ATCC, USA) and Raw 264.7 (mouse macrophage, ATCC, USA) were cultured in Dulbecco's modified Eagle's medium (DMEM, Gibco, USA) supplemented with 10% fetal bovine serum (FBS, Gibco, USA) and 1% penicillin/streptomycin (P/S, Gibco, USA). These cultures were maintained in a 5% CO_2_ incubator at 37 °C. THP1‐Dual and THP1‐Dual KO cells (RIG‐I KO, MAVS KO, MDA5 KO, cGAS KO, STING KO cells, Invivogen, Hong‐Kong) were cultured in RPMI 1640 media (Gibco, USA) supplemented with 10% fetal bovine serum (FBS, Gibco, USA), 1% penicillin/streptomycin (P/S, Gibco, USA), and selective antibiotics. These cells were also maintained in a 5% CO_2_ incubator at 37 °C, following the manufacturer's instructions. Raw‐Dual cells (Invivogen, Hong‐Kong) were cultured in DMEM supplemented with 10% fetal bovine serum, and 1% penicillin/streptomycin.

### Protein Expression In Vitro

To assess protein expression using IVT mRNAs, HEK 293T cells (2 × 10^4^ cells per well), HeLa cells (1 × 10^4^ cells per well), or Raw 264.7 (5 × 10^4^ cells per well) were seeded in 96‐well plates and allowed to grow overnight. Subsequently, 70 ng of IVT mRNA samples were transfected into the plated cells using Lipofectamine 2000 or LNP formulation. After 24 h post‐transfection, protein expression efficiency was measured. For the IVT mRNA samples encoding firefly luciferase, the protein expression was quantified following the manufacturer's protocol for the Bright‐Glo Luciferase Assay System (Promega, USA). To evaluate protein expression using IVT mRNAs encoding red fluorescent protein (RFP), the transfected cells were lysed with CelLytic M (100 µL per well, Sigma–Aldrich, USA) for RFP expression measurement using a microplate reader (Excitation 530 nm, Emission 580 nm). To assess spCas9 protein expression using IVT mRNAs and ACE mRNA, Raw 264.7 cells (5 × 10^5^ cells mL^−1^) were seeded in 6‐well plates and allowed to grow overnight. Two micrograms of either IVT mRNA or ACE mRNAs were transfected to the plated cells using Lipofectamine 2000. At 24 h post‐transfection, transfected cells were harvested, and the efficiency of spCas9 protein expression was measured by western blot analysis.

### Type I IFN Assay Using Wild‐Type THP1‐Dual, THP1‐Dual KO cells, or Raw‐Dual Cells

THP1‐Dual and THP1‐Dual KO cells (7.2 × 10^4^ cells per well) were plated in a 96‐well plate and induced to differentiate into macrophage‐like cells using 25 ng mL^−1^ of phorbol 12‐myristate 13‐acetate (PMA, Invivogen, Hong Kong) following the manufacturer's guidelines. After 3 h of PMA treatment, the cells were rinsed with 1X PBS and cultured in fresh media for 72 h. Subsequently, they were treated with various 200 ng mRNA samples (unmodified IVT mRNA, Ψ‐modified IVT mRNA, or ACE mRNA (mRNA/DNA25B)), as well as various control substances to validate immune responses (2′3′‐cGAMP (5 µg mL^−1^, Invivogen, Hong Kong), 3p‐hpRNA (300 ng mL^−1^, Invivogen, Hong Kong), or human IFN‐β (104 IU mL^−1^, Abcam, UK)) for 24 h. Following this treatment, the induction of type I IFN responses was assessed using the Quanti‐Luc reagent (Invivogen, Hong Kong) in accordance with the manufacturer's instructions. Raw‐Dual cells (1 × 10^5^ cells per well) were plated in a 96‐well plate and allowed to grow overnight. Subsequently, they were treated with 200 ng of various mRNA samples (unmodified IVT mRNA, Ψ‐modified IVT mRNA, or ACE mRNAs, as well as various control substances to validate immune responses (2′3′‐cGAMP (10 µg mL^−1^, Invivogen, Hong Kong) and VACV‐70/LV (1 µg mL^−1^, Invivogen, Hong Kong) for 24 h. Following this treatment, the induction of type I IFN responses was assessed using the Quanti‐Luc reagent (Invivogen, Hong Kong) following the manufacturer's instructions.

### Animals

Female BALB/c mice (6 weeks old) were obtained from KOATECH (South Korea). All animal experiments were approved by the Institutional Animal Care and Use Committee of Gyeongsang National University (Protocol number: GNU‐230214‐M0029‐01) and the Institutional Animal Care and Use Committee of Korea Advanced Institute of Science and Technology (IACUC of KAIST) (Protocol number: KA2022‐074‐v2).

### EPO ELISA

Female BALB/c mice (6 weeks old) were utilized to verify the EPO expression efficiency of the various IVT mRNA samples in vivo. The mice (*n* = 3) were subjected to retro‐orbital venous sinus injection^[^ ^48^, ^49^, ^50^ ^]^ (injection volume: 100 µL) under isoflurane anesthesia, receiving one of the following treatments: unmodified IVT mRNA/LNP, Ψ‐modified IVT mRNA/LNP, or ACE mRNA/LNP (mRNA/DNA25B or mRNA/DNA65C). The control group was injected with 1X PBS. At the 3, 6, 9, and 18 h post‐injection time points, blood samples were collected, and plasma was isolated via centrifugation at 3000 RCF for 10 min at 4 °C.

To quantify EPO levels in the plasma, an EPO sandwich ELISA (Enzyme‐linked immunosorbent assay) was employed. The process involved coating a 96‐well immune plate with capture EPO antibodies, followed by blocking with 5% BSA buffers. Subsequently, the collected plasma samples were added to the antibody‐coated and blocked plate and allowed to incubate. After washing with 0.05% PBST, a biotinylated detector antibody was applied and incubated. Further, 100 µL of streptavidin conjugated to HRP was introduced to the plate and incubated for 30 min. After another wash with 0.05% PBST, a TMB substrate solution (Thermo Scientific, USA) was added and allowed to incubate until a color change occurred. The reaction was stopped with 100 µL of stop solution (Thermo Scientific, USA), and the EPO levels were quantified by measuring the absorbance at a wavelength of 450 nm using a microplate reader (VICTOR Nivo, Perkin Elmer, USA).

### Statistical Analysis

Statistical analysis was performed using GraphPad Prism 6 statistical software (Student's *t*‐test), and the *p*‐value was adjusted for statistical significance. (n.s: not significant, *p < 0.05, **p < 0.01, and ***p < 0.001, *n* = 3). Data are reported as means ± standard deviation.

## Conflict of Interest

The authors declare no conflict of interest.

## Supporting information

Supporting Information

## Data Availability

The data that support the findings of this study are available from the corresponding author upon reasonable request.
