# Supplementary material for: ACE mRNA (Additional Chimeric Element incorporated IVT mRNA) for Enhancing Protein Expression by Modulating Immunogenicity
Source: Adv Sci (Weinh). 2024 Mar 6;11(18):2307541. doi: 10.1002/advs.202307541 (PMC11095206; doi:10.1002/advs.202307541)
Supplement: Supplementary file 1 — Supporting Information [file ADVS-11-2307541-s001.pdf]

ADVANCED SCIENCE
Open Access

# Supporting Information

for *Adv. Sci.*, DOI 10.1002/advs.202307541

ACE mRNA (Additional Chimeric Element incorporated IVT mRNA) for Enhancing Protein Expression by Modulating Immunogenicity

*Sora Son, Minsa Park, Jin Kim and Kyuri Lee**

Supporting Information

**ACE mRNA (Additional Chimeric Element incorporated IVT mRNA) for Enhancing Protein Expression by Modulating Immunogenicity**

*Sora Son, Minsa Park, Jin Kim, Kyuri Lee**

College of Pharmacy and Research Institute of Pharmaceutical Sciences, Gyeongsang National University, Jinju, Gyeongsangnam-do 52828, Republic of Korea

E-mail: leekyuri@gnu.ac.kr

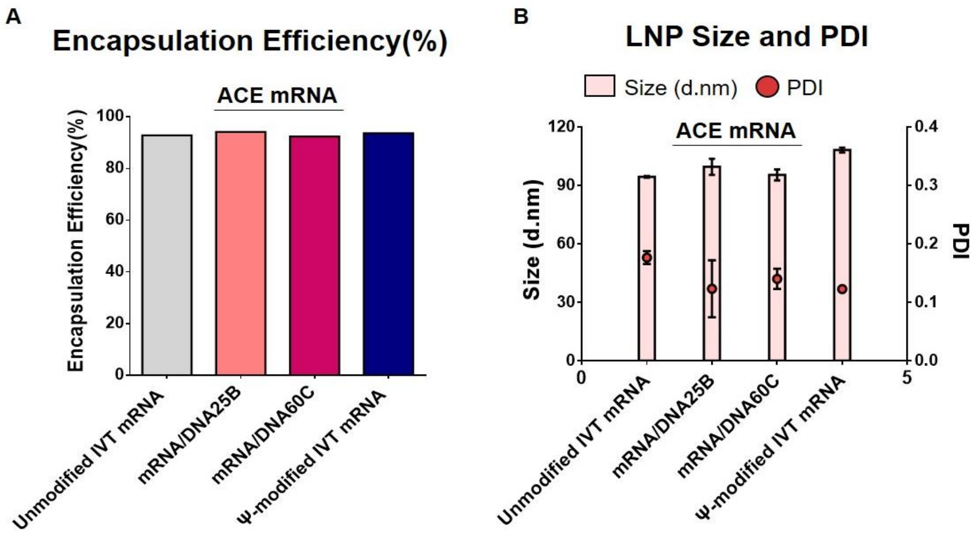


**Figure S1. Verification of LNP encapsulating RFP encoded IVT mRNA samples (unmodified IVT mRNA, Ψ-modified IVT mRNA, and ACE mRNA (mRNA/DNA25B, mRNA/DNA60C).** A) The encapsulation efficiency (%) of all mRNA constructs was measured by Ribogreen assay. B) The hydrodynamic size and PDI of all mRNA were measured using dynamic light scattering (DLS).

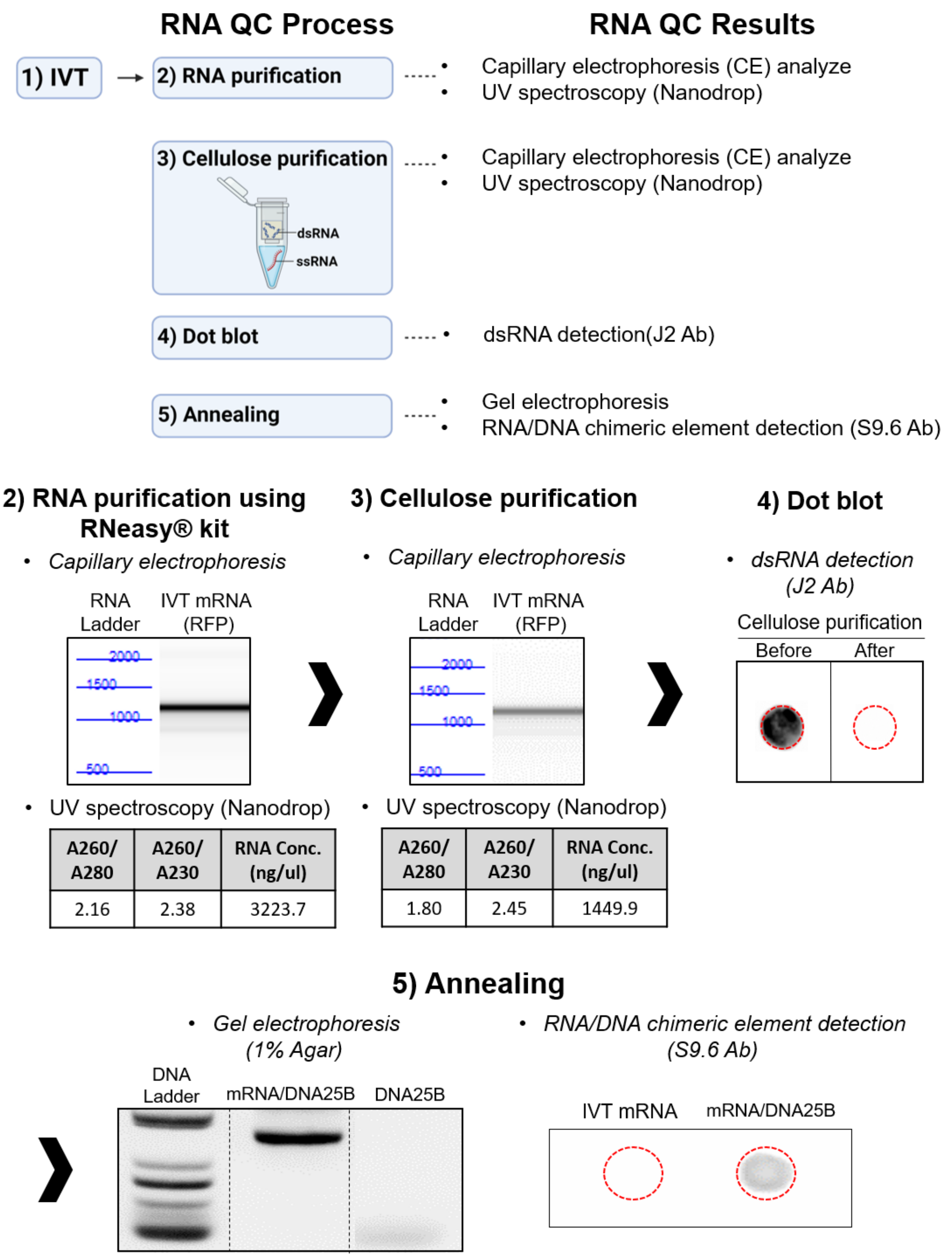


**Figure S2. The comprehensive procedure for the preparation of ACE mRNA.** The manufacturing process and workflow of ACE mRNA. 1) IVT mRNAs with or without additional sequences were synthesized by in vitro transcription (IVT) using EZ™ MEGA T7 Transcription kit. 2) IVT mRNA was purified using the RNeasy® Kit, and RNA quality was assessed by capillary electrophoresis analysis and UV spectroscopy. 3) Cellulose purification was used for the removal of dsRNA by-products, and RNA quality was assessed by capillary electrophoresis analysis and UV spectroscopy. 4) The dot blot assay was performed for the

measurement of dsRNA before and after cellulose purification. 5) After confirmation of dsRNA removal, DNA oligonucleotides with sequences complementary to the additional sequences were annealed to the in vitro transcribed mRNA (IVT mRNA) containing those additional sequences, resulting in the additional RNA/DNA chimeric element incorporated mRNA (ACE mRNA). The presence of RNA/DNA chimeric elements within the IVT mRNA was detected using the S9.6 antibody.

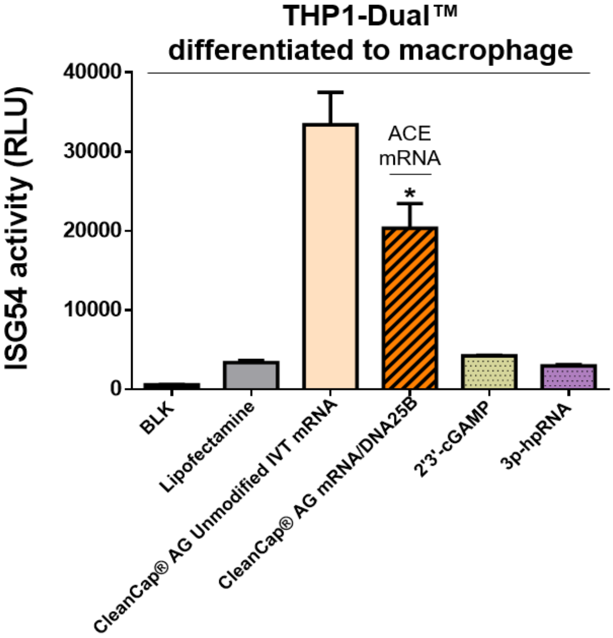


**Figure S3. The immunomodulatory effects of ACE mRNA prepared with CleanCap® AG.** The CleanCap® AG cap (Trilink) was utilized in the preparation of ACE mRNA, followed by measurement of type I IFN responses using THP1-Dual™ cells. ACE mRNA prepared with CleanCap® AG (CleanCap® AG mRNA/DNA25B) showed reduced ISG54 activity compared to its counterpart unmodified IVT mRNA prepared with CleanCap® AG (CleanCap® AG Unmodified IVT mRNA).

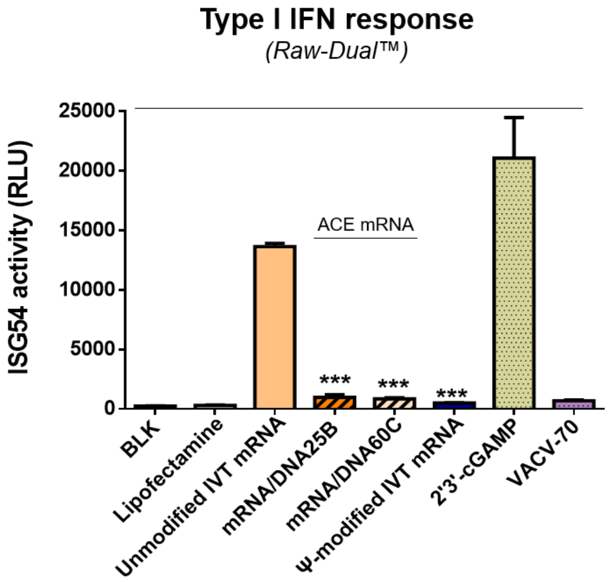


**Figure S4. Evaluation of type I IFN responses of ACE mRNA in Raw-Dual™ cells.** The type I IFN responses induced by ACE mRNA were verified in Raw-Dual™ cells. In comparison to cells treated with unmodified IVT mRNA, Raw-Dual™ cells treated with ACE mRNA exhibited a decrease in ISG54 activity.

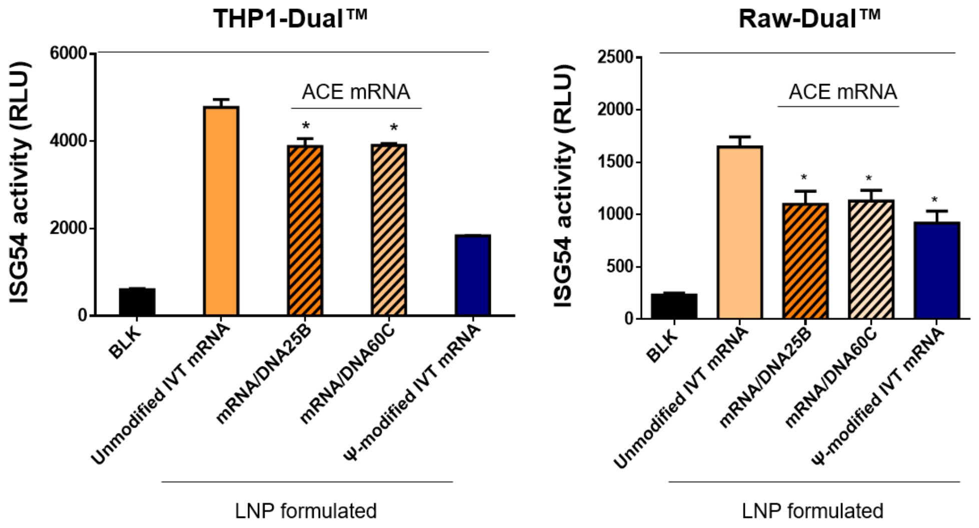


**Figure S5. Evaluation of type I IFN responses induced by ACE mRNA formulated in the LNP system.** The type I IFN responses of ACE mRNA formulated in LNP system were evaluated using THP1-Dual™ and Raw-Dual™ cells. Both ACE mRNA variants (mRNA/DNA25B or mRNA/DNA60C) formulated in the LNP system showed reduced type I IFN responses compared to the LNP-formulated unmodified IVT mRNA.

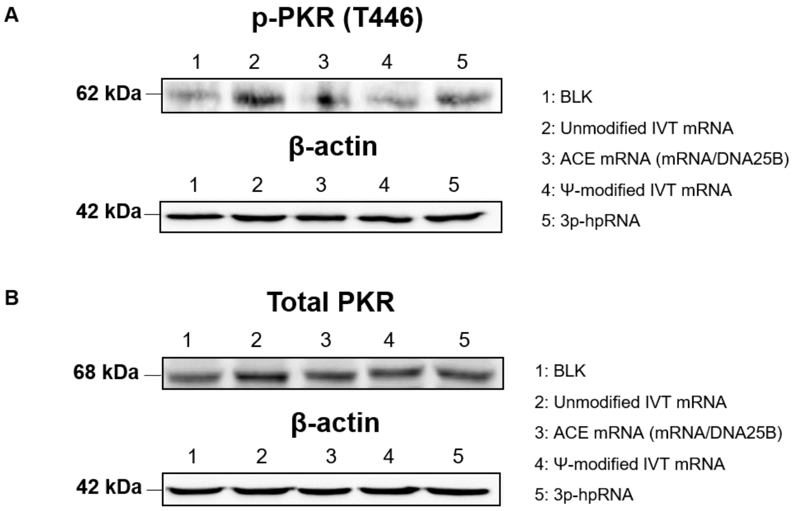


C

**Relative Band density**
(p-PKR/Total PKR)

| BLK | Unmodified IVT mRNA | ACE mRNA (mRNA/DNA25B) | Ψ-modified IVT mRNA | 3p-hpRNA |
|---|---|---|---|---|
| 0.26 | 1.00 | 0.65 | 0.48 | 0.98 |

**Figure S6. Western blot analysis to examine the cellular PKR expression and phosphorylation**

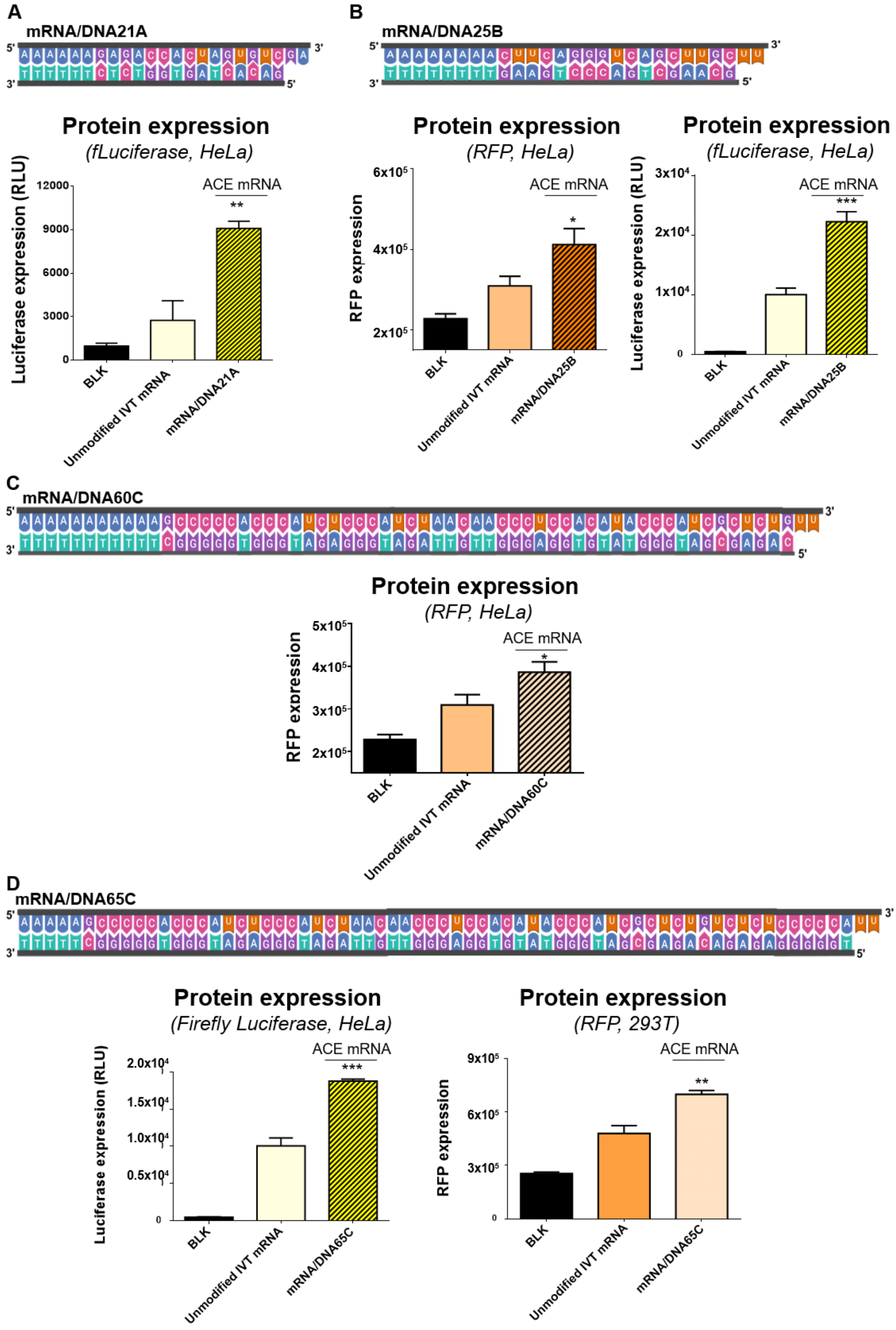


**Figure S7. The ACE mRNA with distinct designs of introduced RNA/DNA chimeric elements**

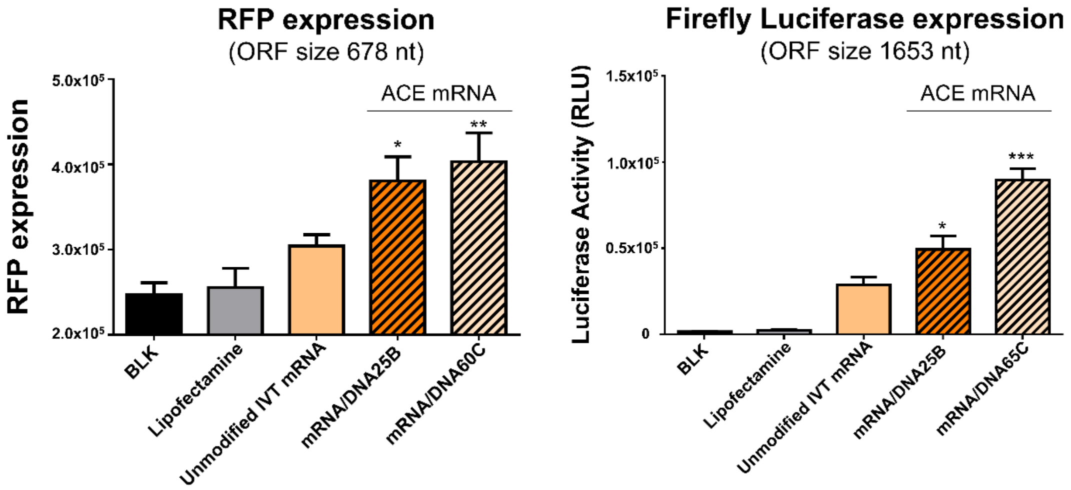


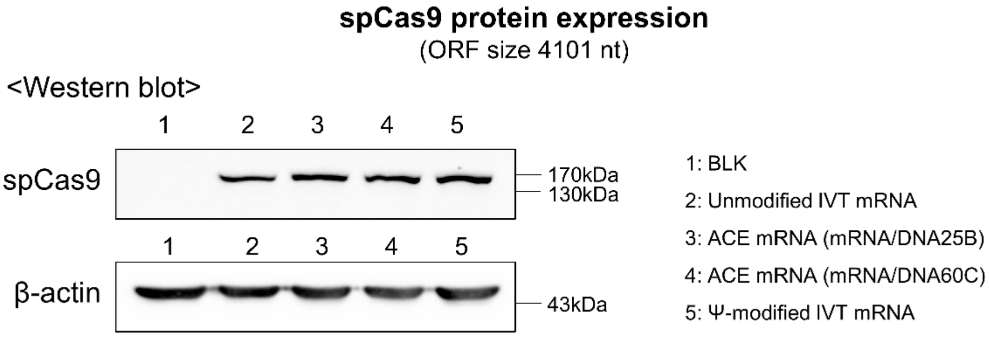


**Relative Band Adj. Volume**
(Cas9/β-actin)

| BLK | Unmodified IVT mRNA | ACE mRNA (mRNA/DNA25B) | ACE mRNA (mRNA/DNA60C) | Ψ-modified IVT mRNA |
|---|---|---|---|---|
| 0.0 | 0.17 | 0.26 | 0.26 | 0.36 |

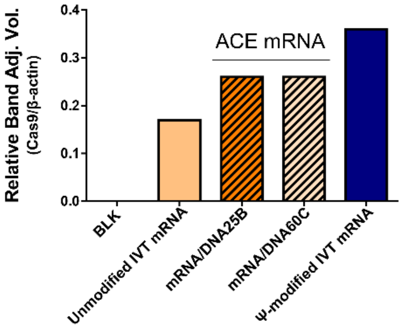


**Figure S8. Protein expression efficiency of ACE mRNA encoding different target proteins.**

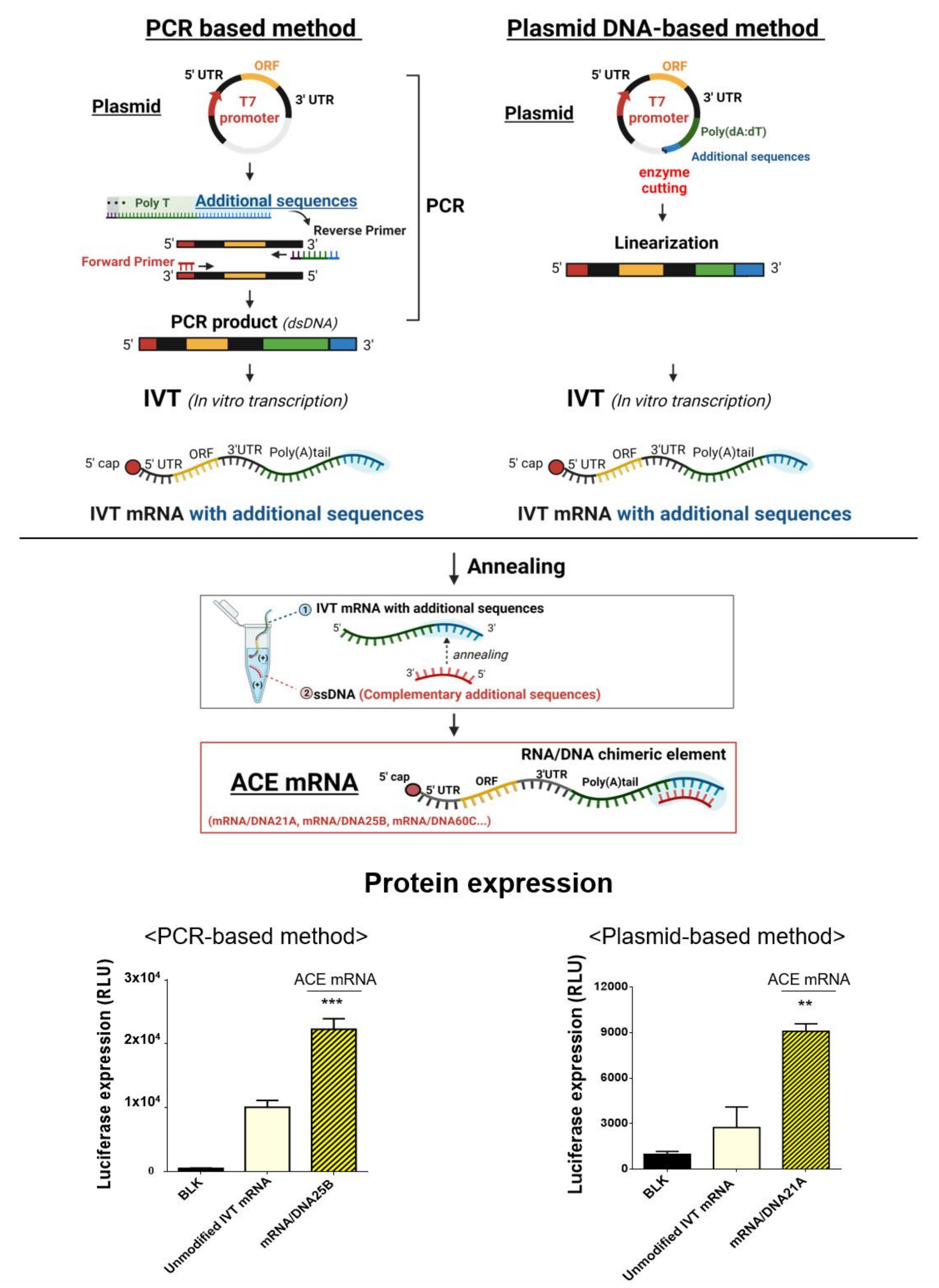


**Figure S9. Comparison between PCR-base and plasmid-based methods in the preparation of ACE mRNA.**

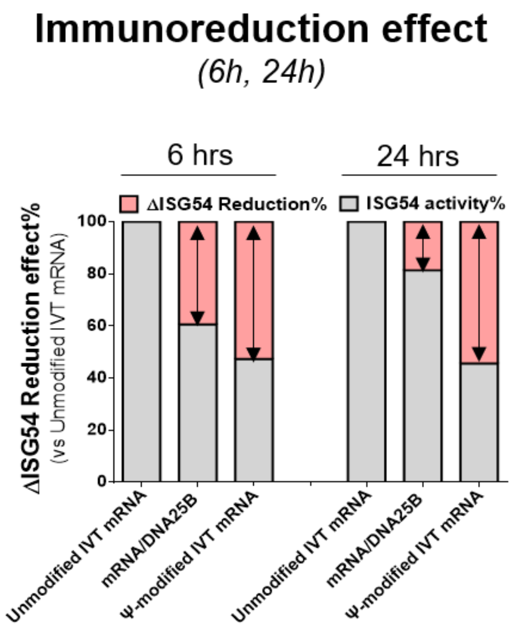


**Figure S10. Evaluation of the immunoreduction effect of ACE mRNA in THP1-Dual™ cells at different time points (6 hrs and 24 hrs post-treatment).**
